# *CHROMOMETHYLASE3* governs male fertility to affect seed production in tomato

**DOI:** 10.1093/hr/uhaf143

**Published:** 2025-05-29

**Authors:** Huihui Zhu, Weiwei Chen, Zheng’an Yang, Liang Chen, Li Huang, Yiguo Hong, Jianli Yang

**Affiliations:** Key Laboratory of Vegetable Biology of Yunnan Province, College of Landscape and Horticulture, Yunnan Agricultural University, No. 452, Fengyuan Road, Panlong District, Kunming 650201, China; College of Life and Environmental Sciences, Hangzhou Normal University, No. 2318, Yuhangtang Road, Xihu District, Hangzhou 311121, China; Key Laboratory of Vegetable Biology of Yunnan Province, College of Landscape and Horticulture, Yunnan Agricultural University, No. 452, Fengyuan Road, Panlong District, Kunming 650201, China; CAS Key Laboratory of Plant Germplasm Enhancement and Specialty Agriculture, Wuhan Botanical Garden, The Innovative Academy of Seed Design, Chinese Academy of Sciences, No. 201, Jiufeng Road, East Lake New Technology Development Zone, Wuhan, China; Laboratory of Cell & Molecular Biology, Institute of Vegetable Science, Ministry of Agriculture Key Laboratory of Horticultural Plant Growth, Zhejiang University, No. 866, Yuhangtang Road, Xihu District, Hangzhou 310058, China; College of Life and Environmental Sciences, Hangzhou Normal University, No. 2318, Yuhangtang Road, Xihu District, Hangzhou 311121, China; Worcester-Hangzhou Joint Molecular Plant Health Laboratory, School of Science and the Environment, University of Worcester, Henwick Grove, Worcester WR2 6AJ, UK; Key Laboratory of Vegetable Biology of Yunnan Province, College of Landscape and Horticulture, Yunnan Agricultural University, No. 452, Fengyuan Road, Panlong District, Kunming 650201, China

## Abstract

To produce mature seed, flowering plants must undergo successful male and female gametogenesis and pollination followed by fruit set, growth, and ripening. This sequential process involves complex genetic programming and less understood epigenetic reprogramming. Here we report a previously unidentified *CHROMOMETHYLASE3*-directed epi-control in pollen mother cell (PMC)-to-microspore transition that determines male fertility to affect seed formation. We generated and characterized hairpin RNA-mediated RNAi and CRISPR/Cas9 transgenic tomato lines in which *CHROMOMETHYLASE3* (*CMT3*) was either knockdown (KD) or knockout (KO). *CHROMOMETHYLASE3* has pleiotropic effects on vegetative and reproductive growth, including leaf, flower, and seed development, besides its influence on tomato ripening and fruit size. However, CMT3 KD plants exhibited stronger effects than KO plants in terms of these vegetative and reproductive processes. Real-time quantitative PCR analysis suggested that genetic compensation might contribute to the less impact of KO plants on pollen and seed development. Integrated RNA-seq and whole-genome bisulfite sequencing reveal that *CMT3* functions as an epi-switch via a self-feedback mechanism to modulate gene expression and governs early development of microspores from PMCs prior to the tetrad stage during microsporogenesis to microgametogenesis, possibly through the pectin catabolic process, to establish pollen fertility that affects seed production in tomato.

## Introduction

Seed production in ripe fruits is vital for angiosperms to survive and thrive under constantly changing climates. Seed starts to form, develop, and mature after successful male and female gametogenesis and pollination; in most flowering plants, this is coupled with fruit setting, growth, and ripening. This complex sequential process is genetically programmed [1–3] and involves epigenetic mechanisms, including RNA-directed DNA methylation (RdDM) of cytosine (^m^C) in CG, CHG, and CHH (where H is A, C, or T) contexts and ^m^C maintenance [4, 5]. In *Arabidopsis* and other species, *de novo* RdDM is established by DOMAINS REARRANGED METHYLTRANSFERASE2 (DRM2) and its homologs [6] with the contribution from DRM1 [7]. METHYLTRANSFERASE1 (MET1) alongside VARIANT IN METHYLATION proteins, or the plant-specific CHROMOMETHYLASE3 (CMT3), together with the H3K9-specific methyltransferase KRYPTONITE, is responsible for maintenance of ^m^CG [8] or ^m^CHG and to a lesser extent ^m^CHH [9, 10], respectively. DRM2 and CMT2 contribute to maintain ^m^CHH [11, 12]; particularly, CMT2 prefers the asymmetric CHH sites [13].

Defects in plant RdDM and ^m^C maintenance affect gametogenesis, seed, and fruit development [14–16]. For instance, a null *MET1* mutation impairs rice seed production, and all seedlings germinated from maldeveloped seeds suffer instantaneous necrotic death despite no phenotypic changes in gametophytes [16]. In *Arabidopsis*, however, homozygous *met1* mutants exhibit a high variation in flowering time and epigenetic diversification of gametes due to loss of ^m^CG maintenance, which is mainly maternally inherited [14]. *MET1*-knockdown (KD) or knockout (KO) also suppresses seed production in tomato and promotes vivipary in rice and the tomato epimutant *Colourless non-ripening* (*Cnr*) [17, 18]. *Arabidopsis drm1*, *drm2*, and *rdr2* (*RNA-dependent RNA polymerase2*) mutants lead to loss of male sexual-lineage–specific RdDM, which causes mis-splicing of the *MULTIPOLAR SPINDLE1* pre-mRNA and meiotic disruption in meiocytes despite no deleterious impact on pollen and seed development [19]. In contrast, rice *AGO2*-KD produces abnormal anther and initiates premature tapetum programmed cell death (PCD) and pollen abortion [20]. All these mutants are associated with abnormal genome-wide ^m^C changes, although global ^m^C alternations also occur dynamically during formation of male and female gametophytes and development of fruit and seed in wild-type plants [4, 21]. However, the precise relevance of such reversible epi-modification or remodeling to vegetative and reproductive growth and the pertinent underlying mechanisms are largely unknown.

In flowering plants, pollen is generated by pollen mother cells (PMCs) and nonreproductive anther primordium cells within anthers through spatiotemporally coordinated events of microsporogenesis to microgametogenesis [22]. During microsporogenesis, PMCs undergo meiosis I and II to produce haploid tetrad microspores that are located within the center of the anther and surrounded by epidermal, cortical, and tapetal cell layers, which arose from nonreproductive cells. Released uninucleate microspores then go through microgametogenesis via pollen mitosis I and II to form bi/trinucleate mature microgametophytes [23]. In this article, we report that *CMT3* affects microsporogenesis at the initial stage of pollen development and imposes epi-effects on male fertility, which affects seed production.

## Results

### *SlCMT3* modulates vegetative and reproductive growth

**Figure 1:**
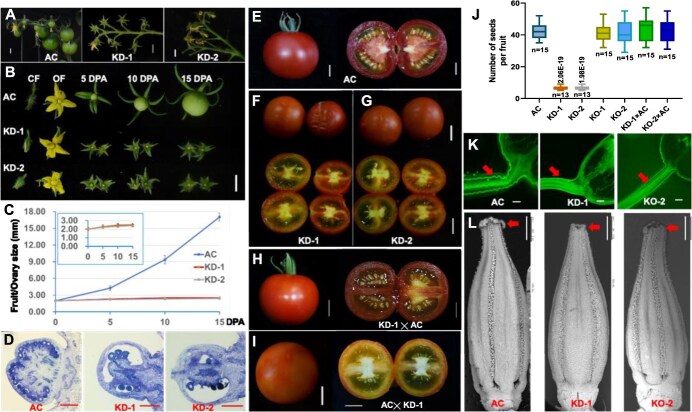
*SlCMT3* controls pollen fertility and seed production. (**A**) Fruits on trusses of wild-type *S. lycopersicum* cv. Ailsa Craig (AC) and two independent *SlCMT3*-KD lines KD-1 and KD-2. (**B**) Flowers and fruits at different developmental stages. Wild-type and *SlCMT3*-KD closed flower (CF) at 3 days prior to anthesis and opened flower (OF) at 0-day postanthesis (DPA) are shown. Wild-type and *SlCMT3*-KD fruits at 5, 10, and 15 DPA are presented. (**C**) Size of wild-type and *SlCMT3*-KD expansion-stalled fruits (ovaries). The inset graph shows the similar size of ovaries of the two KD lines. (**D–G**) Impact of *SlCMT3* on early seed abortion in ovary (**D**) and on seed production in fruits of wild-type AC (**E**), *SlCMT3*-KD lines KD-1 (F), and KD-2 (**G**). (**H, I**) Effect of *SlCMT3* on seed production in *SlCMT3*-KD x AC (H) and reciprocal AC x *SlCMT3*-KD (I) cross-fruits. (**J**) Number of mature seeds in tomato fruits. *P* values of Student’s *t*-tests between AC and KD lines and number of fruits (n) are indicated. (**K**) AC pollen forms pollen tubes (arrow) in AC, *SlCMT3*-KD or *SlCMT3*-KO styles. (**L**) Impact of *SlCMT3* on production of pollen grains around the tips (arrow) of anther cones. Bar = 1 cm in Panels A, B, and E–I; bar = 500 μm in Panel D; bar = 1 mm in Panels K and L. Data are mean ± SD (n = 13–15 biological replicates). Data statistical analyses were performed by Tukey’s test analysis of variance, and statistically significant differences were determined at the *P* ≤ 0.05 level.

We previously demonstrated that *SlCMT3* is required to maintain hypermethylation at the *epi-*locus responsible for the colorless nonripe fruit phenotype in the *Cnr* epimutant [24–26]. This prompted us to explore how *SlCMT3* controls ripening in wild-type tomato. To achieve this, we generated 12 independent *SlCMT3*-KD lines in *Solanum lycopersicum* cv. Ailsa Craig (AC, Supplementary Table S1) via a gene-specific RNAi strategy (Supplementary Fig. S1A) [17, 27]. Compared to AC, endogenous *SlCMT3* transcripts were reduced by 63%–97% in KD leaf tissues (Supplementary Table S1; Supplementary Figs S1B, S2A, and  S3A). *SlCMT3*-KD affected vegetative leaf development, reproductive flower function, or seed production (Fig. 1A–C; Supplementary Figs S1, S2B–I, and  S3B–F), in addition to its effect on fruit size and ripening (Supplementary Fig. S4A–D). Based on these diverse phenotypes, the 12 *SlCMT3*-KD lines were classified into three groups (Supplementary Table S1). Group I, comprising eight independent lines exhibiting developmental abnormalities in leaf morphology, displayed an 84%–97% KD efficiency of *SlCMT3* transcripts in foliar tissues. Floral morphology and fruit setting were not visibly affected. However, less than 8.5% flowers were fertile, and most of the set fruits failed to expand, resulting in the production of only a limited number of small ripening-delayed fruits containing an ~86% reduced number of mature seeds (Fig. 1D–J, Supplementary Fig. S4A, B, D). Indeed, seed abortion and/or nonpollinated ovules in Group I lines were evident in growth-stalled fruits at 0-day postanthesis (DPA; Fig. 1D). Group II, comprising two independent transgenic lines exhibiting pleiotropic developmental defects (including malformed leaves and nonviable floral organs), demonstrated ~75% KD efficiency of endogenous SlCMT3 transcripts in foliar tissues. These lines completely failed to set fruit or viable seeds, ultimately proving nonviable through their entire life cycle. (Supplementary Fig. S2E–K). Group III, comprising two independent transgenic lines demonstrating 63%–67% KD efficiency of endogenous SlCMT3 transcripts in foliar tissues, exhibited fully retained developmental competence. These lines maintained vegetative architecture and reproductive viability indistinguishable from wild-type AC controls, suggesting a threshold effect in SlCMT3-mediated epigenetic regulation (Supplementary Fig. S3B–F). These results indicated that RNAi efficacy is directly proportional to developmental aberration severity.

We also generated homologous CRISPR/Cas9 *SlCMT3*-KO lines, where *SlCMT3-*targeted gene editing resulted in nucleotide deletions (Supplementary Fig. S5A) and no production of SlCMT3 protein, in comparison to only a reduced level of SlCMT3 protein in *SlCMT3*-KD plants (Supplementary Fig. S5B). However, we found *SlCMT3*-KO only partially phenocopied *SlCMT3*-KD in terms of affecting compound leaf development (Supplementary Fig. S5C–I). Unlike Group I and Group II *SlCMT3*-KD lines, *SlCMT3*-KO caused less impact on fruit size (Supplementary Fig. S4A, C, D) and floral/pollen development (Fig. 1K–L; also see below), and no defect in fruit ripening (Supplementary Fig. S4C) and seed development (Fig. 1J; Supplementary Fig. S6A–H). Such differences between *SlCMT3*-KD and *SlCMT3*-KO plants might be ascribed to genetic compensation, considering the importance of CMT3-mediated DNA methylation to plant growth and development. To verify this notion, we analyzed the gene expression of all CMTs in *SlCMT3*-KD and *SlCMT3*-KO plants. Indeed, we found the *SlCMT2* and *SlCMT4* expressions were increased in *SlCMT3* KO line, but no significant difference was found in the *SlCMT3* KD line, when *SlCMT3* expression was decreased in both KO and KD lines compared with AC plants (Supplementary Fig. S6I–J).

Taken together, these results reveal a *SlCMT3-*directed mechanism that controls not only fruit development and ripening, but also vegetative and reproductive growth, including leaf and floral organogenesis, and seed production in tomato. Using Group I *SlCMT3*-KD lines KD-1 and KD-2, and *SlCMT3*-KO lines KO-1 and KO-2, we went on to investigate how *SlCMT3* affects tomato gametogenesis to dictate seed production.

### *SlCMT3* controls pollen fertility

To establish which gamete was impacted by *SlCMT3* and subsequently affected seed production, we crossed *SlCMT3*-KD with AC through fertilizing the stigma of emasculated *SlCMT3*-KD flowers with wild-type AC pollen grains. The resulting pollinated *SlCMT3*-KD flowers were almost 100% fertile and produced normal ripening fruits in which seeds developed normally (Fig. 1H). By contrast, the reciprocal AC x *SlCMT3*-KD cross caused abscission of AC flowers 2–4 days after artificial fertilization. Of more than 100 emasculated AC flowers that were hand pollinated with *SlCMT3*-KD pollen, only very few led to the formation of small ripe fruits in which no seed formed and matured properly (Fig. 1I and J). Such infertility was not observed in AC x *SlCMT3*-KO and reciprocal *SlCMT3*-KO with AC crosses (Supplementary Fig. S6G and H). We also noted that male and female floral organs in AC, *SlCMT3*-KD and *SlCMT3*-KO were morphologically similar although the lengths of stamen and style were 15–18% shorter than AC in *SlCMT3*-KD (Supplementary Fig. S1F–I), and this was also seen to a lesser degree in *SlCMT3*-KO than AC (Supplementary Fig. S5F–I). This did not affect pollination and seed production since AC pollen was able to elongate and form pollen tubes inside styles after fertilization onto *SlCMT3*-KD or *SlCMT3*-KO stigma (Fig. 1K), and indeed normal fruits and seeds developed (Fig. 1H, Supplementary Fig. S6H). However, compared to AC, fewer pollen grains appeared around the tips of anther cones in *SlCMT3*-KD and *SlCMT3*-KO lines (Fig. 1L). Despite this the KO lines do not seem to show any reduced sterility as seed production is not affected (Fig. 1J). We interpret these data to mean that the sterility in *SlCMT3*-KD lines is due to male but not female infertility, and that defect in *SlCMT3-*directed methylation remodeling during the male gametophyte development causes male sterility and thereby inhibition of seed production, fruit-set or/and fruit growth. This suggests that a functional *SlCMT3* is required for proper male gametogenesis (pollen development) and fertility, leading to apt ovule pollination and subsequent formation of viable seeds in tomato.

### SlCMT3 governs early pollen development

**Figure 2:**
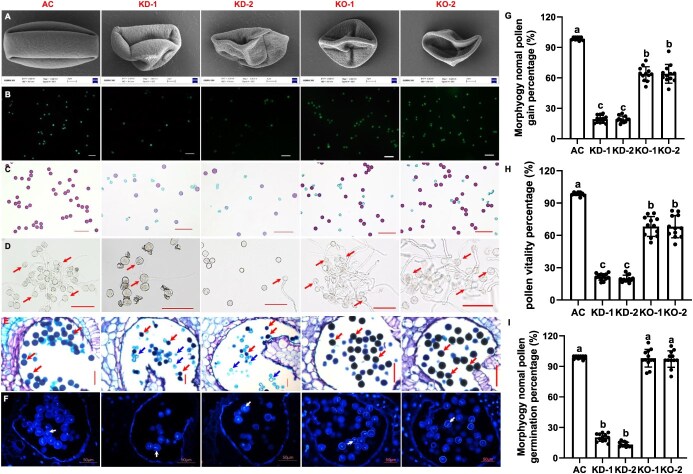
Influence of *SlCMT3* on pollen morphology and viability. (**A**) SEM examination of pollen grains. Pollen grains were collected from 0-DPA flowers of AC, *SlCMT3*-KD lines KD-1 and KD-2, and *SlCMT3-KO* lines KO-1 and KO-2. (**B**) FDA staining. Live pollen grains (AC > KO > KD) show green fluorescence. (**C**) Alexander Red staining. Live pollen grains (AC > KO > KD) show red while dying and dead pollen grains (KO > KD > AC) stain blue. (**D**) *Ex vivo* pollen germination assay. Live pollen grains (AC > KO > KD) can germinate and pollen tubes can be seen. Examples of germinating pollen grains are indicated by red arrows. (**E**) Paraffin sectioning of anthers. Anthers were collected from 0-DPA flowers of AC, *SlCMT3*-KD lines KD-1 and KD-2, and *SlCMT3*-KO lines KO-1 and KO-2. Paraffin sections were stained with toluidine blue. Mature viable pollen grains show deep-blue staining (red arrows), while dying or dead pollen grains do not. (**F**) DAPI staining. Bright blue fluorescence shows mature pollen grains are binuclear (AC > KO > KD). Examples of binuclear pollen grains are indicated by white arrows. Bar = 3 μm (A), 100 μm (B–D), or 50 μm (E and F). (G–I) Percentage of normal pollen (G), pollen viability (H), and germinated pollen (I) collected from 0-DPA flowers of AC, *SlCMT3*-KD lines KD-1 and KD-2, and *SlCMT3-KO* lines KO-1 and KO-2. Pollen was collected from 12 different flowers at 0 DPA. Data are mean ± SD (n = 12 biological replicates). Lowercase letters above the bars represent statistically significant difference at *P* ≤ 0.05 level.

To define at which pollen developmental stages *SlCMT3* initiated its impacts on male fertility, we examined diploid sporogenous PMCs (i.e. meiocytes, also known as microsporocytes), haploid microspores, and mature pollen grains in AC, *SlCMT3*-KD, and *SlCMT3*-KO plants. First, we checked mature pollen grains collected from flowers at 0 DPA (Fig. 2A–F, Supplementary Fig. S7A–D) and found that the portion of shrunken abnormal pollen (Fig. 2A, Supplementary Fig. S7A) increased significantly in *SlCMT3*-KD (average ~80%) or *SlCMT3*-KO (average ~32%) compared to AC (less than 1.5%) (Fig. 2G, Supplementary Fig. S7B). *Ex vivo* pollen viability and germination assays showed that about 98% AC pollen grains were viable and able to germinate (Fig. 2B–D). For *SlCMT3*-KD, ~20% were alive and only 13% capable of germinating (Fig. 2H and I). The viability of *SlCMT3*-KO pollen was reduced by a lesser extent to 67–68%, and of these viable pollen grains 97–98% were able to germinate (Fig. 2H and I; Supplementary Fig. S7C and D). *In situ* transverse anther sections revealed that viable pollen grains were predominant in AC anther sac, while fewer appeared in *SlCMT3*-KO and the fewest in *SlCMT3*-KD. Dead pollen grains and those lacking nuclei were evident in *SlCMT3*-KD and *SlCMT3*-KO (Fig. 2E and F; Supplementary Fig. S8). These results establish that *SlCMT3*-KD, and to a lesser extent *SlCMT3*-KO, affects the production of viable mature pollen, and proper male fertility.

**Figure 3:**
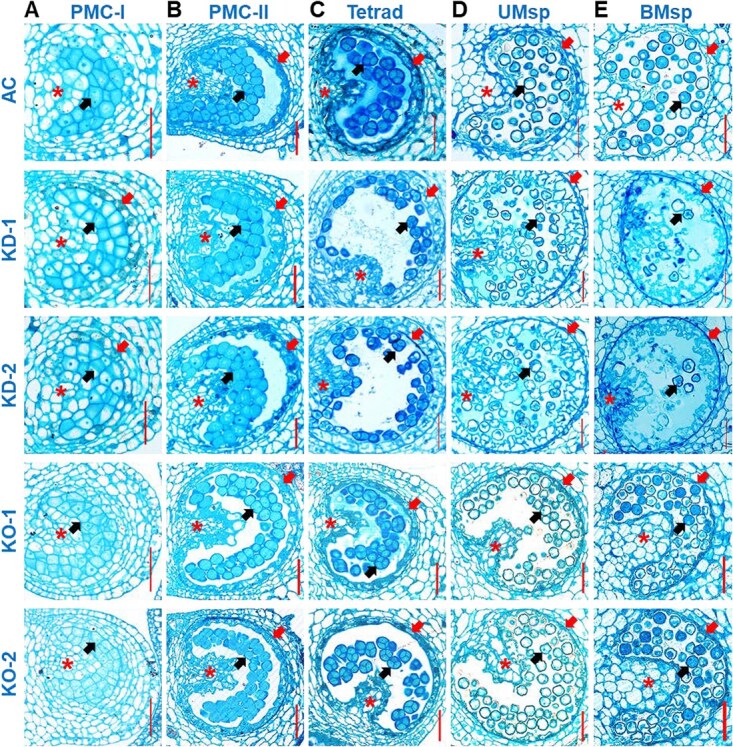
*SlCMT3* influences microsporogenesis and microgametogenesis at different stages of pollen development. (**A–C**) Microsporogenesis—PMCs undergo meiosis I and II to form haploid microspores. PMC stage I (PMC-I, **A**), II (PMC-II, **B**), and Tetrad microspore stage (**C**) are indicated. (**D, E**) Microgametogenesis—uninucleate (UMsp) and binucleate (BMsp) microspores after mitosis I and II to produce mature pollen grains. Wild-type AC, *SlCMT3* RNAi-KD lines KD-1 and KD-2, and *SlCMT3* CRISPR/Cas9-KO lines KO-1 and KO-2 are shown. Asterisk indicates diploid PMC-generating cells; red arrow for tapetum (a layer of diploid cells); and black arrow for PMCs, microspores, or pollen grains. Bar = 50 μm.

**Figure 4:**
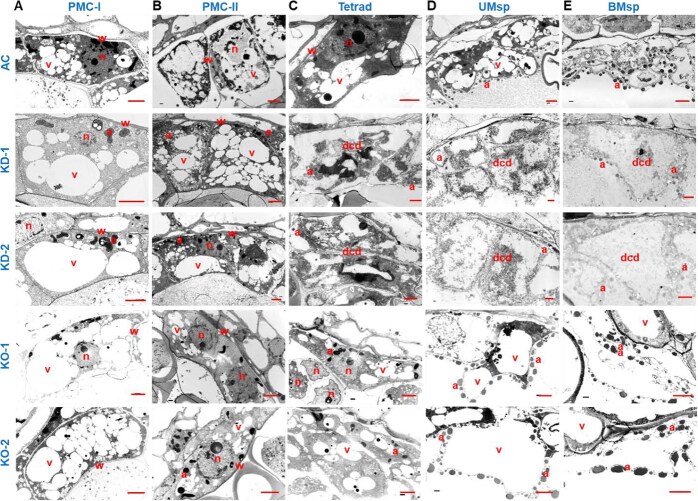
*SlCMT3* defines tapetal cell fate during pollen development. (**A**) Tapetal cells at PMC stage I (PMC-I). (**B**) Tapetal cells at PMC stage II (PMC-II). (**C**) Tapetal cells at tetrad microspore stage (Tetrad). (**D**) Tapetal cells at uninucleate microspore stage (UMsp). (**E**) Tapetal cells at binucleate microspore stage (BMsp). Wild-type AC, *SlCMT3* RNAi-KD lines KD-1 and KD-2, and *SlCMT3* CRISPR/Cas9-KO lines KO-1 and KO-2 are shown. Lowercase letters a, dcd, n, v, and w represent apoptotic body, dead cell debris, nucleus, vacuole, and cell wall, respectively. Bar = 3 μm.

By examining anther cross-sections (Fig. 3), we found that PMCs at PMC-I and PMC-II were indistinguishable between AC and *SlCMT3*-KO; however, a clear layer of tapetum emerged outside PMCs in *SlCMT3*-KD as early as at PMC-I (Fig. 3A). At PMC-II (after meiosis I), the tapetum started to disintegrate and lose regular cell morphology in *SlCMT3*-KD (Fig. 3B). Numerous tetrad microspores collapsed in *SlCMT3*-KD, whereas they appeared normal in *SlCMT3*-KO and AC at the tetrad stage (after meiosis II) (Fig. 3C). On advancing to the uninucleate and bi/trinucleate stages, deformed and inactive microspores were abundant in *SlCMT3*-KD, and irregular pollen grains were also noticeable in *SlCMT3*-KO, while mature microgametophytes developed properly in AC (Fig. 3D and E). Morphologically distorted tapetum and broken tapetal cells occurred at PMC-II onward in *SlCMT3*-KD, and to a lesser extent in *SlCMT3*-KO, but these only happened at the uni-/binucleate microspore stages in AC (Fig. 3C–E). These changes were further confirmed by TEM examination of the ultrastructure of tapetum, microspores, and mature pollen, in which uncharacteristically enlarged vacuoles and apoptotic bodies associated with tapetal cell death could be traced to PMC-I in *SlCMT3*-KD (Fig. 4A–E). Moreover, alongside the morphological, cytostructural, and ultrastructural anomalies, early PCD occurred in tapetum at PMC-I/II in *SlCMT3*-KD while PCD was initiated later at the tetrad stage in *SlCMT3*-KO, and lagged even further in AC, evidenced by increasing occurrence of small fragmented apoptotic bodies (Fig. 4A–E). The meiosis process of PMC in *SlCMT3*-KD and *SlCMT3*-KO plants was basically normal. However, more highly vacuolated cytoplasm of PMC was found in *SlCMT3*-KD and *SlCMT3*-KO from PMC-II stage, and obvious degeneration of cytoplasm was observed adjacent to callose wall (Fig. 4). Although the cytokinesis of meiosis was normal, gradual disintegration of cytoplasm occurred in tetrads and increased afterward (Fig. 4), leaving the microspore almost empty at the early uninucleate microspore stage (Fig. 4). The formation of pollen wall was also blocked in *SlCMT3*-KD and *SlCMT3*-KO as evidenced by the absence of distinct tectum and columellae at the surface of uninucleate microspore. Finally, deformed microspores with residues inside the cell and incompact sporopollenin at the surface were found at the binucleate microspore stage (Fig. 4).

**Figure 5:**
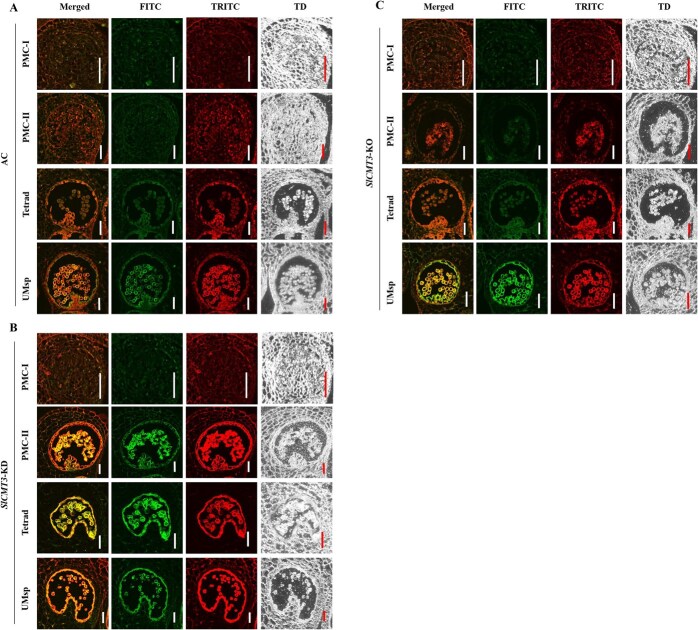
*SlCMT3* affects PCD of PMCs and tapetal cells during early pollen development. TUNEL staining indicates occurrence of PCD at PMC-I, PMC-II, tetrad (Tetrad), or uninucleate (UMsp) microspore stages. (**A**) Wild-type AC. (**B**) RNAi *SlCMT3*-KD line. (**C**) CRISPR/Cas9 *SlCMT3*-KO line. Photographs were taken through transmission detector (TD), TRITC (562 nm), or FITC (488 nm) channels to show the overall morphology of pollen sac, red autofluorescence, and PCD-specific green fluorescence. The TRITC–FITC merged images appear yellow or orange, distinctive to cells undergoing PCD. Bar = 50 μm.

Our spatiotemporal analysis of PCD dynamics revealed striking epigenetic regulation of tapetal degradation schedules. TUNEL-based quantification of programmed DNA fragmentation demonstrated premature PCD initiation in *SlCMT3-KD* anthers, showing DNA breaks detectable as early as PMC-II. While AC exhibited controlled DNA fragmentation initiating at tetrad stage, *SlCMT3-KO* displayed intensified PCD signals at uninucleate microspore (UMsp) stage (Fig. 5A–C). We further revealed that *SlCMT3* encodes a nucleus-localized protein (Supplementary Fig. S9A and B).

These collective data show that *SlCMT3* is required to direct formation of viable microspores from diploid PMCs at the initial stage of male gametogenesis, consistent with the strong *SlCMT3* promoter activity in floral male organ tissues (Supplementary Fig. S9C–J) and its spatiotemporal expression profiles where the level of *SlCMT3* transcripts was most elevated in male meiocyte cells at the PMC-I stage, then gradually decreased to the lowest in mature pollen grains during the progression from microsporogenesis to microgametogenesis (Supplementary Fig. S9K and L).

### SlCMT3 is an epi-switch for PMC-tetrad transition

To elucidate how *SlCMT3* regulates microsporogenesis, we performed integrative analyses of genome-wide datasets generated from Illumina transcriptome-wide RNAseq and whole-genome bisulfite sequencing (WGBS) on duplicated anther/stamen (microspore/pollen) samples collected from two different AC and *SlCMT3*-KD plants at 0 DPA (Supplementary Table S2). The 0-DPA time point exhibited maximal transcriptional divergence between AC and *SlCMT3*-KD, coinciding with phenotypic manifestation windows, which allowed mechanistic dissection of how threshold-level *CMT3* suppression (≤25% residual expression) triggers downstream epigenetic dysregulation. Intriguingly, *SlCMT3*-KD caused an ~4% overall increase in genome-wide ^m^C, mainly in the CG and CHG sites (Supplementary Fig. S10).

**Figure 6:**
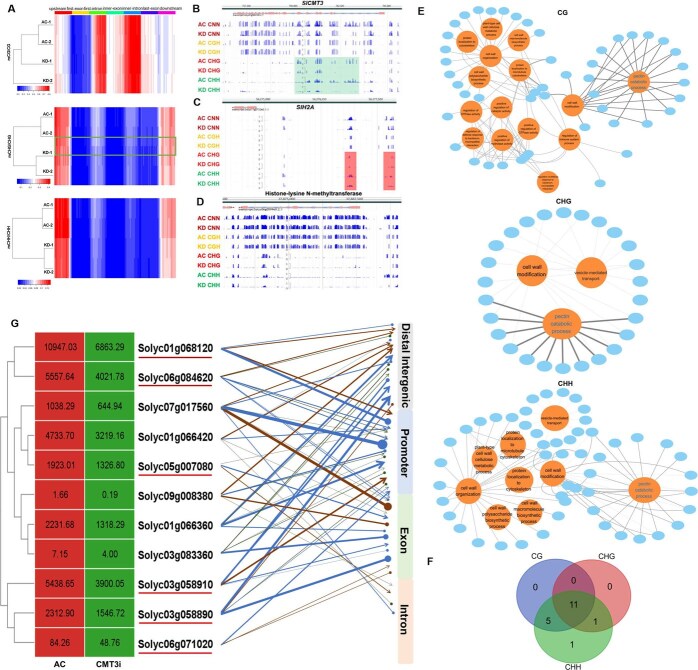
*SlCMT3* epi-modulates pollen development and seed production. (**A**) DNA methylation levels of three ^m^C contexts between AC and RNAi *SlCMT3*-KD line at different genomic regions. (**B–D**) DNA methylation landscape for *SlCMT3* (**B**), *SlH2A* (**C**), and encoding histone-lysine N-methyltransferase gene (**D**) in AC and KD. (**E**) Gene ontology (GO) enrichment analysis of biological process for these DEG^DMR^s at CG, CHG and CHH. (**F**) Venn diagram of DEG^DMR^s in pectin catabolic process. (**G**) The correlation of 11 overlapped gene expression in F and its DNA methylation at different gene region. The highest DNA hypermethylation or hypomethylation level at the same region were selected as representation. The arrow represents hypermethylation and the circle represents hypomethylation. Blue, brown, and green lines indicated CG, CHG, and CHH methylation, respectively.

Bioinformatics mining WGBS datasets led to the identification of 284 510, 266 995 and 599 974 genes with differentially methylated regions (DMRs) in the ^m^CG, ^m^CHG, or ^m^CHH context, respectively (Supplementary Table S3-S5). Overall, the DNA methylation level exhibited different tendencies between AC and *SlCMT3*-KD plants at 0 DPA, especially at CHG context (Fig. 6A). In addition, we also identified 1819 differentially expressed genes (DEGs) from the comparative ‘AC vs *CMT3*-KD’ transcriptomes (Table S2). To further examine the interconnection between methylation changes and gene expression, we correlated WGBS and transcriptomics analyses. In addition, 1464, 1228, 1621 DEGs with ^m^CG, ^m^CHG, or ^m^CHH-type of DMGs were identified, respectively, which occupied almost all DEGs in the comparative ‘AC vs *CMT3*-KD’ transcriptomes (Table S3-S5). These results revealed the changes in DEGs were mostly dependent on DNA methylation change. All these DEGs are designed DEG^DMR^s thereafter. Defects in these DEG^DMR^s have been shown to impact on cell cycle and division, mitotic progression, PCD, cell wall establishment, actin and cytoskeleton, cutin biosynthesis, receptor kinase signaling and vesicle trafficking to affect tapetal cell fate, pollen and anther exine formation, male gametogenesis, pollen fertility, cross- and self-incompatibility, pollen tube growth and attraction, signaling in male–female gamete interactions, or embryogenesis and seed viability (Table S3–S5). To our surprise, *SlCMT3* was identified as a DEG^DMR^ in *SlCMT3*-KD vs AC comparison and had hypo-DMRs with a clear reduction of ^m^CHG and ^m^CHH that were mapped to the *SlCMT3* introns and exons in *SlCMT3*-KDs (Fig. 6B and Table S5). In addition, DEG^DMR^s with hypo-DMRs were found to encode H2A and the histone-lysine N-methyltransferase that methylates H3K4 and H3K36 (Fig. 6C and D and Table S3). Both genes are associated with chromatin architecture and epigenetic chromatin remodeling [4], specifically expressed in male gametic cells, and affecting pollen development [28]. Consistently, using real-time quantitative PCR (RT-qPCR), we also identified differential expression of genes known to be linked with establishing tapetum, meiosis, PCD, and pollen development in both KD and KO lines (Supplementary Fig. S11). These results revealed SlCMT3-mediated DNA methylation regulated gene expression cooperated with chromatin architecture and remodeling to establish pollen development.

To obtain a global overview of these DEG^DMR^s, we performed a GO enrichment analysis of biological process. Interestingly, the most significantly enriched category was identified for the pectin catabolic process in these DEG^DMR^s, whether at CG, CHG, or CHH (Supplementary Fig. S12 and Fig. 6E), indicating pectin was vital for the SlCMT3-mediated pollen development process. Meanwhile, most of these DEG^DMR^s at three contexts were overlapped and found DNA methylation simultaneously occurred at CG, CHG, or CHH in 11 DEG^DMR^s (Fig. 6F). Moreover, these 11 DEG^DMR^s were downregulated and high DNA methylation level occurred at CG context (Fig. 6G). Besides, most of them underwent DNA methylation at distal intergenic regions and promoter regions, indicating DNA methylation repressed gene expression by influencing chromatin structure and its transcriptional regulation (Fig. 6G). In *Arabidopsis*, *VANGUARD1* (*VGD1*) homologous to a pectin methylesterase (PME) gene was reported to be vital for pollen development via modifying cell wall [29]. Pectate lyase-like proteins (PLLs) are required for intine loosening during the first steps of pollen tube germination [30]. Notably, of 11 DEG^DMR^s, 6 DEG^DMR^s were homologies of PMEs (Solyc01g068120 and Solyc06g084620) and PLLs (Solyc03g058890, Solyc03g058910, Solyc05g007080, and Solyc06g071020) (Fig. 6G). These results indicated DNA methylation-mediated gene expression involved in the pectin catabolic process might participate in pollen development and its fertility.

**Figure 7:**
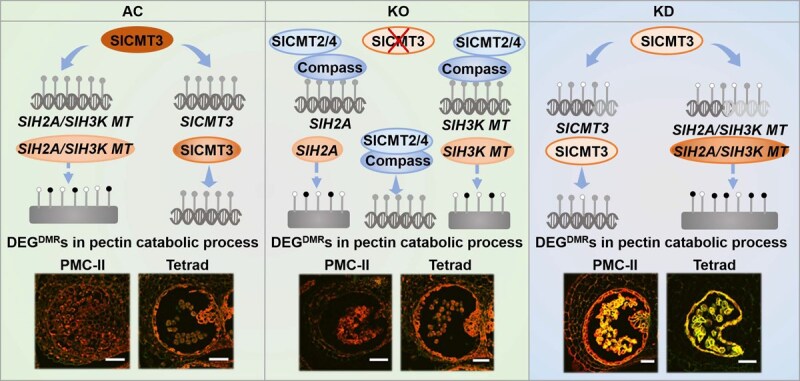
A model for *SlCMT3*-mediated epigenetic control in the pollen development process. In AC plants, SlCMT3-mediated DNA and histone methylation functions as an epi-switch governing pollen development. H2A and histone-lysine N-methyltransferase (H3K MT) are incorporated into the epi-pathway. The gray double helix indicated DNA methylation and rectangle indicated histone methylation. In *SlCMT3* KO line, the unknown genetic compensation (Compass) is involved to offset *SlCMT3* as highlighted in blue. In *SlCMT3* KD lines, the reduced function of SlCMT3 and lack of compass resulted in improper methylation, which caused deregulation of a group of genes implicated in pectin catabolic process and defective in pollen development.

Taken together, our data illustrate that *SlCMT3* may act as an epi-switch to modulate expression of *DEG^DMR^*s, including *SlCMT3* itself, and affect expression of other pollen development-related genes. This may then define the kinetics of tapetum development and the timing of PMC-tetrad transition during microsporogenesis to microgametogenesis, possibly via the pectin catabolic process, to establish pollen fertility that affects seed production in tomato (Fig. 7).

## Discussion

Male gametogenesis in plants involves complex genetic and transcriptional regulatory networks [31, 32] and extensive epigenetic reprogramming [4]. Such epi-reprogramming can be achieved at different stages of pollen development through chromatin remodeling via changed levels of H1, H2A.Z, H3K4me3, H3K9me2, and H3K27me3 [4, 33, 34]; dynamic DNA methylation by differential expression of *AGOs*, *DCL1/4*, *RDR2*, *DDM1*, *DRM1/2*, *MET1* (*MET1a/b*), *CMT2/3*, *SUVH5*, *DME*, *ROS2*, and *DML2/3* [4, 35, 36]; and small RNA cross-talking between vegetative and sperm cells within mature pollen grains [4, 37–40]. However, the biological significance of such epi-reprogramming is often unknown. Here, we define a *CMT3*-directed epi-mechanism in pollen fertility through an epi-effect on PMC-tetrad microspore transition that affects seed production.

Given the RNAi effect of up to a 97% reduction of *SlCMT3* transcript in vegetative leaf tissues and mature pollen grains, the influence on *SlCMT3* mRNA levels from PMC-I to the later uninucleate stage, particularly the significant increase at the PMC-II stage during male gametogenesis in *SlCMT3*-KD lines, was unexpected. These findings suggest that the hairpin *SlCMT3*-dsRNA can trigger RNAi-mediated *SlCMT3*-KD in vegetative leaf tissues to affect leaf architecture and in mature pollen grains, but it exerts an opposite influence on *SlCMT3* expression in reproductive cells to govern early PMC and microspore development (Supplementary Fig. S6I). This may be due to hypomethylation-induced transcriptional activation of *SlCMT3* via a self-feedback mechanism (Figs 6B and 7). Moreover, the milder to nondeleterious effect on pollen and seed (and fruit) development in *SlCMT3*-KO lines suggests that an unknown genetic compensation mechanism [41] may be able to off-set the effects of a completely dysfunctional *SlCMT3* gene that has lost its capacity to express SlCMT3 protein in *SlCMT3-*KO tomato (Fig. 7; Supplementary Fig. S5B).

While the dosage compensation hypothesis (*CMT2*/*CMT4* functional redundancy) provides a plausible explanation for the WT-like phenotype in *CMT3*-KO lines, our experimental evidence delineates distinct regulatory paradigms in KD variants. Three key observations challenge a universal compensation model: (1) Nonlinear phenotypic penetrance emerges in KD groups despite comparable CMT2/CMT4 expression profiles across all lines (Fig. S6J). (2) Threshold-dependent phenotypic manifestation occurs specifically in Group I/II lines with >75% *CMT3* suppression, uncorrelated with compensatory methyltransferase activity. (3) Group III’s attenuated phenotype (63–67% *CMT3* reduction) aligns with RNAi efficiency thresholds rather than compensatory upregulation. This triphasic response pattern suggests two distinct regulatory tiers: (i) subcritical *CMT3* suppression (<70%) permits developmental buffering through residual CMT3 activity, and (ii) supracritical KD (>75%) overwhelms homeostatic capacity, unmasking dosage-sensitive pathways independent of paralog compensation. These findings necessitate revisiting the compensation hypothesis, proposing instead a kinetic threshold model where phenotypic outcomes depend on CMT3 activity falling below critical thresholds rather than compensatory network activation. Future studies employing temporal-resolved methylome profiling could further disentangle these mechanisms.

Furthermore, the *SlCMT3*-directed epi-control in male fertility differs from the *MET1*-mediated epi-influence on female gametogenesis to affect seed formation [7, 14], although *MET1* is also implicated to play a role in pollen reprogramming during embryogenesis and PCD of brassica and tobacco tapetal cells [41]. *Arabidopsis DRM1/2*- and *RDR2*-mediated RdDM-specific male linkage, which affects PMC meiosis but imposes no detrimental impact on pollen and seed production [19], is clearly different from *SlCMT3*-KD, which causes severe pollen defects leading to seed abortion in tomato. However, whether and how *SlCMT3-KD* affects seed development *per se* remains to be elucidated. Although both *CMT3* and *AGO2* can affect tapetum PCD, unlike *AGO2*-KD that induces tapetal cells to undergo PCD after the tetrad stage in rice [20], *SlCMT3*-KD triggers tapetum formation as early as at the PMC-I stage and induces early PCD of PMCs and tapetal cells during the initial process of microsporogenesis, accompanied by DNA methylation regulation.

In addition, RNA-seq and WGBS integration revealed that *CMT3-*mediated DNA methylation modulated pectin catabolic process to govern pollen development (Figs 6 and 7). Pectin functions as the main component of the pollen wall, especially influenced by the fine-tuning of pectin methylesterase activity [42]. However, most of the previous research focused on gene function instead of gene regulation with respect to pollen development. In our study, we found that DNA methylation in pectin catabolic process-related genes regulated their transcriptional change, and thereby modulated pollen development. This offers a promising strategy for DNA methylation modification to regulate fruit development and improve crop production.

In conclusion, our results demonstrate that a *CMT3* self-feedback pathway controls pollen fertility that affects seed production. This is achieved via an epigenetic influence on the early transition from PMCs to microspores as well as the fate of tapetal cells, possibly via pectin catabolic process, which provides a unique route to generate male sterile lines for tomato and other crop breeding in the future.

## Materials and methods

### Plant materials and growth conditions

*Solanum lycopersicum* cv. Ailsa Craig (AC), *Nicotiana benthamiana*, and *Arabidopsis thaliana* Col-0 were used in this study. A nontranslatable 275-bp fragment was cloned into the pRNAi-LIC vector [17] to generate RNAi lines, *SlCMT3*-KD. *SlCMT3*-KO lines were generated by CRISPR/Cas9 technology. A 1.5-kb noncoding promoter region of *SlCMT3* was cloned into pCAMBIA1300-GUSplus vector and pCAMBIA1300/GFP vector to produce p*SlCMT3Pro:GUS* in AC background and p*SlCMT3Pro:GFP* in *A. thaliana* Col-0 background, respectively. Moreover, SlCMT3 coding sequence was the pCAMBIA1300/35S-GFP vector to produce SlCMT3ox lines. Among numerous transgenic tomato and *Arabidopsis* lines, at least two independent homozygous lines for each transformation were used for further genetic, epigenetic, cellular and/or molecular analyses. The growth conditions were the same as the previously described conditions [17].

### Histochemical GUS assay and GFP fluorescence detection

Histochemical GUS assay was performed as described [43]. For subcellular localization, the fluorescent signals of SlCMT3-GFP fusion proteins emitted by leaf cells were photographed via the FITC channel following the Nikon A1’s protocol. Similarly, GFP fluorescence in transgenic *Arabidopsis* SlCMT3Pro:GFP lines, fully opened flowers and pollen grains were also photographed through the FITC channel.

### Pollen and callose wall observation

To investigate *in vivo* pollen germination and pollen tube elongation, pistils were collected at 1-day postpollination/cross, fixed in 2-ml solution containing 1.5-ml 95% ethanol and 0.5-ml glacial acetic acid (v/v 3:1), decolorized in 2-ml 8 M NaOH, washed with sterile water, stained in 0.5% toluidine blue and photographed under Nikon Eclipse Ni epi-fluorescence microscope. For *in vitro* pollen viability assay, pollen grains were collected from anthers that were dissected from floral buds. Pollen viability was then determined with fluorescein diacetate (FDA) staining or Alexander (AD) staining. Pollen viability rate was calculated as percentage of the number of viable pollen out of the total number of pollen grains counted under Nikon Eclipse Ni epifluorescence microscope via the FITC channel or under TD for FDA or AD staining, respectively. *Ex vivo* pollen germination assay was performed by incubating pollen grains collected from flowers on pollen germination medium and then calculated the percentage of the number of germinating pollen with visible pollen tubes out of the total number of pollen grains counted under the same Nikon microscope as pollen germination rate. For examination of callose deposition, aniline blue staining was performed as described [44].

### Electron microscopy observation

Ovaries/ovules and anthers/stamen were taken from tomato floral buds and flowers at different developmental stages. After fixed and embedded, tissue sections were stained in 0.5% toluidine blue or a DAPI (4′,6-diamidino-2-phenylindole) solution to show nuclei of pollen grains and photographed with a Nikon Eclipse Ni epifluorescence microscope. Further, 1% methylene blue staining was applied to examine pollen development at the cytostructural level, floral buds corresponding to the PMC, dyad, tetrad, early and late uninucleate pollen, binucleate pollen, and mature pollen stage. For ultrathin sectioning, the same samples as prepared for semithin sectioning were sectioned to 60-nm specimens using Leica UC7, stained first with uranyl acetate and then with alkaline lead citrate. Photographs were taken using the Hitachi Model H-7650 transmission electron microscope (TEM). To examine tomato pollen ultrastructure, pollen grains collected from unopened floral buds and fully opened flowers were directly spread on to the scanning electron microscopy (SEM) carriers.

### TdT-mediated dUTP nick end labeling assay

Tomato floral buds at different developmental stages were fixed, embedded, and sectioned following the protocol for paraffin-sectioning. After deparaffinization and permeabilization, nick-end labeling of fragmented DNA was performed on the sections using the Fluoresce *In Situ* Cell Death Detection Kit (Roche) following Roche’s guidance. Sections were then examined and photographed via transmission detector (TD), tetramethylrhodamine isothiocyanate (TRITC) (562 nm), or fluorescein isothiocyanate (FITC) (488 nm) channel under a Nikon A1 laser confocal microscope.

### Western blot

To investigate SlCMT3 expression in wild-type, SlCMT3-KD, and SlCMT3-KO transgenic tomato plants, total protein was extracted from tomato leaf tissues as described [43], and a polyclonal anti-SlCMT3 antibody raised against the C-terminal peptide KRKASPADSSSDSSC of the SlCMT3 protein in rabbits (GeneScript, Nanjing, China) was used for immunodetection. Western blot analysis was also performed with a monoclonal anti-actin (plant) antibody (Sigma-Aldrich A0480) to show a comparable level of protein loading for each individual sample.

### RT-qPCR analysis

Total RNA was extracted from leaf, buds, floral organs, and stamens at different developmental stages using either RNAprep Pure Plant Kit (Tiangen) or RNeasy Plant Mini Kit (Qiagen). First-strand cDNA synthesis and the following quantitative PCR were performed as described in our previous study [17] with the gene-specific primer pairs (Supplementary Table S6). At least three technical replicates for each of three biological replicates were performed in at least two repeated experiments.

### RNA sequencing and WGBS

The wild-type and KD tomato stamen tissue samples were used in RNA isolation for RNAseq and extraction of genomic DNA by the DNeasy Plant Mini Kit (Qiagen) for WGBS, respectively. The library construction and data analysis in RNAseq and WGBS were performed as described in our previous study [27]. DEGs were then identified with DMR(s) in the context of CG, CHG, or CHH, respectively, or in combination of CG, CHG, and CHH, named as epiDEG^DMR^.

### Statistical analysis

Quantitative data were generated from at least three biological replicates of wild-type plants and multiple independent transgenic lines in different assays in repeated experiments. Numerical data are represented in the format of mean ± SD (standard deviation). Tukey’s test, one-way ANOVA test, or Student’s *t*-test was performed, wherever appropriate, to analyze if any statistically significant difference between various treatments existed. *P* value less than or equal to 0.05 was regarded to be different with statistical significance.

## Supplementary Material

Web_Material_uhaf143

## Data Availability

All data in the main text and the supplementary materials are available upon request. WGBS and RNA-seq datasets have been deposited in NCBI under accession number PRJNA1175415.
