# Supplementary material for: CHROMOMETHYLASE3 governs male fertility to affect seed production in tomato
Source: Hortic Res. 2025 May 29;12(9):uhaf143. doi: 10.1093/hr/uhaf143 (PMC12313338; doi:10.1093/hr/uhaf143)
Supplement: Web_Material_uhaf143 [file web_material_uhaf143.zip › Supplement Table 1 & 6.pdf]

Table S1 Phenotypical classification of SlCMT3-KD transgenic lines

***CMT3*-KD Group No**	**Transgenic Lines No**	***CMT3*-KD Efficiency***	**Flowers VS Ripe Fruits****	**Pleiotropic Phenotypes**
I	KD-1 (9)	84.53%	290 flowers vs 19 fruits	Abnormal leaf architectures (fig. S1); Morphologically normal flower with no visible defects in floral structure to naked eyes (Fig 1; fig. S1); Fruit set occurred, but only small portion of fruits slowly developed and ripened, and most failed to expand. (Fig. 1, fig. S1; fig. S4)
	KD-2 (10)	97.07%	218 flowers vs 15 fruits	
	KD-3 (3)	85.87%	310 flowers vs 25 fruits	
	KD-4 (5)	91.40%	248 flowers vs 21 fruits	
	KD-5 (7)	89.07%	309 flowers vs 26 fruits	
	KD-6 (8)	89.83%	216 flowers vs 17 fruits	
	KD-7 (11)	86.18%	173 flowers vs 10 fruits	
	KD-8 (12)	86.73%	161 flowers vs 11 fruits	
II	KD-9 (13)	75.39%	201 flowers vs 0 fruits	Severe defects in compound leaf and flower. Abnormal flowers tended to die at 8-10 days after anthesis and no fruit was produced (fig. S2)
	KD-10 (14)	75.37%	237 flowers vs 0 fruits	
III	KD-11 (1)	67.35%	37 flowers vs 35 fruits	No phenotypic changes compared to AC (fig. S3)
	KD-12 (4)	63.63%	30 flowers vs 29 fruits	

***SlCMT3-KD (knockdown) efficiency was calculated as "100% - percentage of the level of SlCMT3 mRNA transcripts in each of the independent SlCMT3-KD line vs that in the control AC line." The SlCMT3 mRNA level was analyzed using RT-qPCR on SlCMT3-KD and AC leaf tissues.**

**Number of flowers with no morphological abnormality visible to naked eyes in Group-I/III lines, but with severe defects in Group-II lines vs number of ripe fruits produced from these 'normal or abnormal' flowers.

Table S6 Primers used in this study.

Primer name	Primer sequence (5'-3')	Gene locus	Application
**For RT-qPCR anlysis:**			
ACTIN-F	AGGCAGGATTTGCTGGTGATGATGCT	Solyc03g078400	Reference gene
ACTIN-R	ATACGCATCCTTCTGTCCCATTCCGA		
18S rRNA-F	CGGCTACCACATCCAAGGAAGG		Reference gene
18S rRNA-R	GAGCTGGAATTACCGCGGCTG		
Ubi3-F	AGAAACCCTAGAAGCCGCAG	Solyc01g056940	Reference genes
Ubi3-R	TGGTGTCGGAAGACTCAACC		
SlCMT3-F	AGTACCAGTTGCCCGGGCTTT	Solyc01g006100	
SlCMT3-R	GAGATTCGTCATTGGAGACCAGTTC		
Histone H4-F	GTGGAGCAAAACGTCATCGTA	Solyc04g011390	Genes involved in meiosis during PMC stage
Histone H4-R	AACACCTCTTGTCTCTTCATAAATC		
Ribosomal-F	CGAGTTCACTCGCTCCCAAA G	Solyc06g075180	
Ribosomal-R	TGGCTTGACGATTCTGCACT		
NuDK-F	AGCCTTCACTCTTGGATCTATG	Solyc01g089970	
NuDK-R	TAAAACCAAACAGGCTAACAGC		
H2A-F	TGGAGGTGTTCTTCCTAACATC	Solyc09g010400	
H2A-R	TTAAGCCTTTCGTGGTGATTTG		
Pho-F	TTGCCTGGATTTACCCCACC	Solyc01g006900	
Pho-R	GATGCTGATAACGCCTCGGA		
Gly-F	CTACAACGATCGCTCTTCAAGA	Solyc01g109660	
Gly-R	GACGAGACCAATCTAAAAAGCC		
60S-F	ATCCTCTGGAGGACCATCCG	Solyc05g053670	
60S-R	CACCTTGAGAGCATCAGGGA		
40S-F	GCGTATTCAGAAGGGACCTGT	Solyc11g006690	
40S-R	AAGGGTCTCCTTGTCGACCT		
AG-like 1-F	ATGAGTCGTCTTCTCAGTTGAG	Solyc07g055920	Genes involved in tapetum establishing and
AG-like 1-R	GCAGAACGTAACTTGTCGATTT		
SlSES-F	GGACGTAGAAGGTCGAGTAAAA	Solyc07g063670	
SlSES-R	TCTGATCTTGTACTCGAAGACG		
EMS1/EXS-F	TGACCTACCCCCTGATGTGT	solyc09g098420	
EMS1/EXS-R	AGATCGATGCACCCAAAGCA		
TPD1-F	GCTCGTCTTATTAACCCTCGTA	Solyc03g097530	
TPD1-R	GCAGACAACAGAAGAAACAGAG		
BAM1/2-F	CCATGCGAGAGGTAGTGCAA	solyc02g091840	
BAM1/2-R	TTGTGTCCCCGGGAATTGAG		
DYT1-like-F	TGGAATTCCCCAGTACCCCA	Solyc02g079810	
DYT1-like-R	TGTTTGGGACCAATGAGCGT		
PDS5-F	CCAAAGCCGAGCAGGACAG	Solyc03g116930	
PDS5-R	ACTCGATACTGTGCTTGATCCTAA		
TDF1-LIKE1-F	GAACGGATAATGATGTGAAGAACCT	Solyc03g113530	
TDF1-LIKE1-R	CTGGTCTAGACATAAATGCACCTTTT		
TDF1-LIKE2-F	TGGTCCCTCAAAAAGCAGGT	Solyc03g059200	
TDF1-LIKE2-R	TGTGAAGCTCAAGGATGCAGT		
bHLH89/91-F	TCCTGCTACTCATCATGTGC	Solyc01g081100	
bHLH89/91-R	AATTCAATCTCCAACGCGGC		
C1A-F	TGCGGACATGACAAATCGAGA	Solyc07g053460	
C1A-R	CCCCATCCTTCCTCCAATCG		
CPK-F	TGACTCGGTGTTTGCACCAT	Solyc06g069220	
CPK-R	CCCTCCCATTATCGTCCACC		
ASP-F	CGATGACTCAATTTACAGCGTT	Solyc08g068870	
ASP-R	ACCTCTTCTGGCAAATACGTAA		

**Construct for *SlCMT3* -KD transgenic lines:**			
pRNAi-SlCMT3-F	cgacgacaagacccTGTTACACGAGCAGAGCC	Solyc01g006100	To generate *SlCMT3* RNAi recombinant vector
pRNAi-SlCMT3-R	gaggagaagagccctGGTGGTAATGTAAGTAATGG		
Intron-F	CGCAAATACGCATACTGTTATCTG		To detect the *PdK* intron gene in
Intron-R	TATATCCCAATGGCATCGTAAA		
**Construct for *SlCMT3* -KO transgenic lines**			
SlCMT3-BsF	ATATATGGTCTCGATTGGCTTCGGATGAAGTAGCGGAAGGGTT	Solyc01g006100	To generate CRISPR/Cas9-induced *SlCMT3* -KO binary vector
SlCMT3-F0	TGGCTTCGGATGAAGTAGCGGAAGGGTTTTAGAGCTAGAAATAGC		
SlCMT3-R0	AACCCTCTTTCTGACCAGACGATTCGCAATCTCTTAGTCGACTCTAC		
SlCMT3-BsR	ATTATTGGTCTCTAAACCCTCTTTCTGACCAGACGATTCGC		
SlCMT3-KO-F	CTAACCTCTCTTACCCATCA	Solyc01g006100	To detect the mutation sites induced by Cas9
SlCMT3-KO-R	AGTGTACTTCAAACCAAACC		
Cas9-F	CACCATCTACCACCTGAGAA		Detection of *Cas9* gene in SlCMT3-
Cas9-R	CGAAGTTGCTCTTGAAGTTG		
Construct for *SlCMT3Pro::GUS* and *SlCMT3Pro::GFP* transgenic lines			
SlCMT3Pro-F	ctctgaaggatccacggtaccGGGCTAGCGTCGAAGAATTTG	Solyc01g006100	To generate *SlCMT3Pro::GUS* and *SlCMT3Pro::GFP*
SlCMT3Pro-R	cctcagatctaccatggtaccTGATGGGTAAGAGAGGTTAGGGAA		
HPT-F	TTCTGCGGGCGATTTGTGTA		Detection of *HPT* gene in transgenic
HPT-R	GGCTTGTATGGAGCAGCAGA		
**For subcellular localization assay of SlCMT3**			
SlCMT3ox-F	acgggggacgagctcggtaccATGTCGAGCAAACGGAAAGC	Solyc01g006100	To generate 35S::SlCMT3::GFP construct
SlCMT3ox-R	tctagaggatccccgggtaccAACCTTATCTAGAGATTCGTCATTGG		
