# Supplementary material for: CHROMOMETHYLASE3 governs male fertility to affect seed production in tomato
Source: Hortic Res. 2025 May 29;12(9):uhaf143. doi: 10.1093/hr/uhaf143 (PMC12313338; doi:10.1093/hr/uhaf143)
Supplement: Web_Material_uhaf143 [file web_material_uhaf143.zip › Supplementary Figures.pdf]

Supplementary Figures

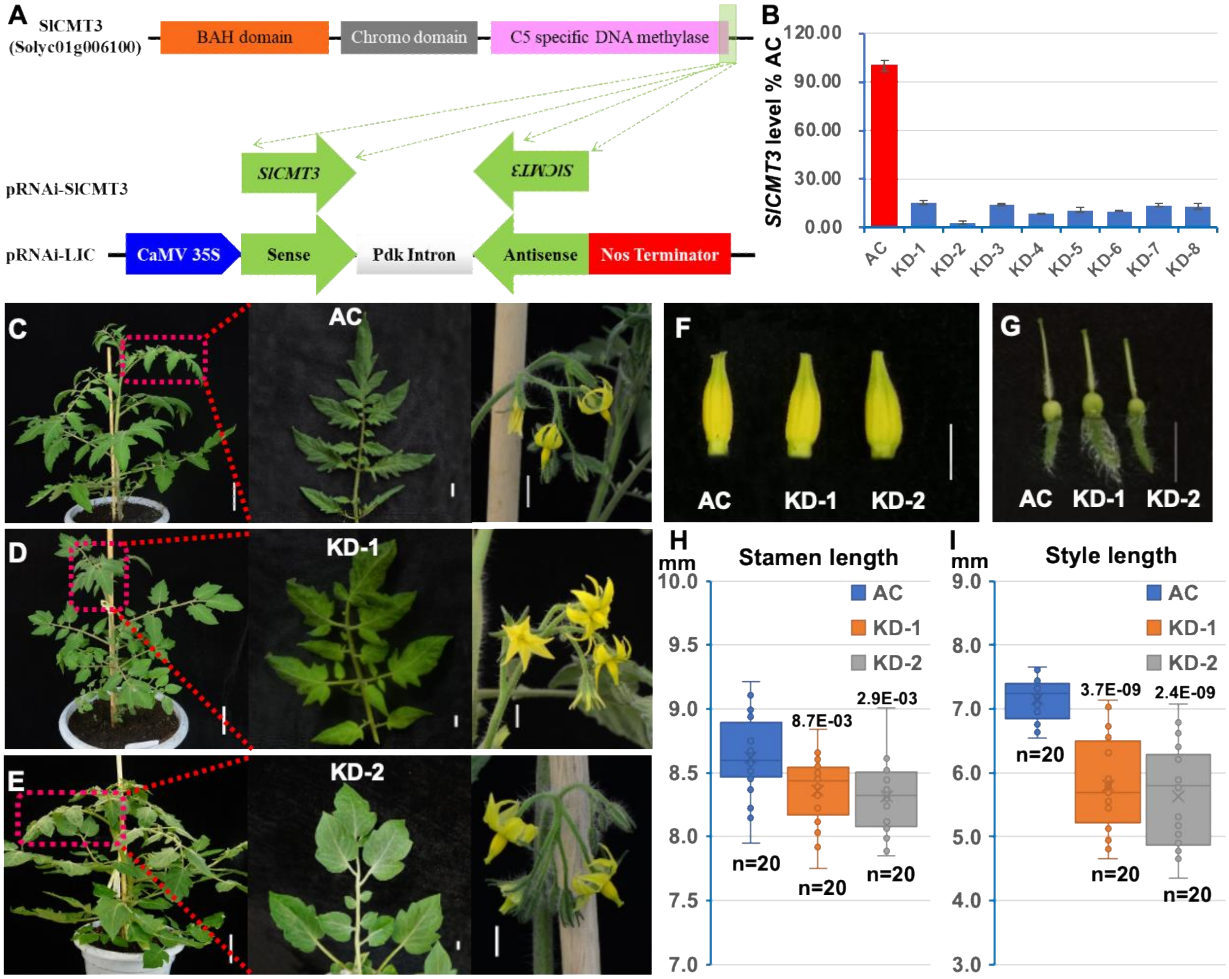

Fig. S1. *SlCMT3*-KD RNAi transgenic tomato lines. (**A**) Construction of pRNAi-SlCMT3. Diagrammatic of *SlCMT3* (Solyc01g006100) with functional domains is shown. A non-translatable 275-bp fragment corresponding to the 3'-portion of the *SlCMT3* mRNA (highlighted, Data set S1) was cloned in both sense and antisense orientations into the pRNAi-LIC vector to produce pRNAi-SlCMT3 as indicated. (**B**) Reduction of *SlCMT3* expression in 8 independent Group-I *SlCMT3*-KD transgenic lines. RNAi-mediated suppression of endogenous *SlCMT3* expression was analysed by RT-qPCR. 18S rRNA was used as the internal control. When compared to the Ailsa Craig (AC) control line transformed with the pRNAi-LIC empty vector, the average level of endogenous *SlCMT3* mRNA transcripts (mean ± SD, n = 4; i.e., four biological replicates/plants per line) was markedly reduced in each of 8 *SlCMT3*-KD transgenic lines KD-1, KD-2, KD-3, KD-4, KD-5, KD-6, KD-7 and KD-8 ($P \leq 0.05$; Tukey's test). Lines KD-1 and KD-2 were used for further investigation.

(**C-G**) Impact of *SlCMT3* RNAi on plant growth and leaf and flower development. In panels C-E, normal vegetative growth of 4-to-5 weeks old plants (left), fully developed normal (C) and abnormal (D, E) compound leaves (middle) and a cluster of flowers at different developmental stages (right) were shown for the control AC line (C) and two *SlCMT3*-KD lines KD-1 (D) and KD-2 (E). Similar morphology of stamen (anther cone, F) and style (G) among the AC control line and two *SlCMT3*-KD lines KD-1 and KD-2. Bar = 5 cm (whole plant) or 1 cm (leaf and flower) in panels C-E, and 5 mm in panels F and G, respectively. (**H, I**) Effect of *SlCMT3*-KD on stamen (anther cone) and style length. Lengths of stamens (H) and styles (I) were measured on 20 fully opened flowers at 0-DPA collected from the AC control line and two *SlCMT3*-KD lines KD-1 and KD-2. Data are shown as mean ± SD (n = 20). Student's ***t***-test was performed on the AC control vs each KD line, and p-values are shown.

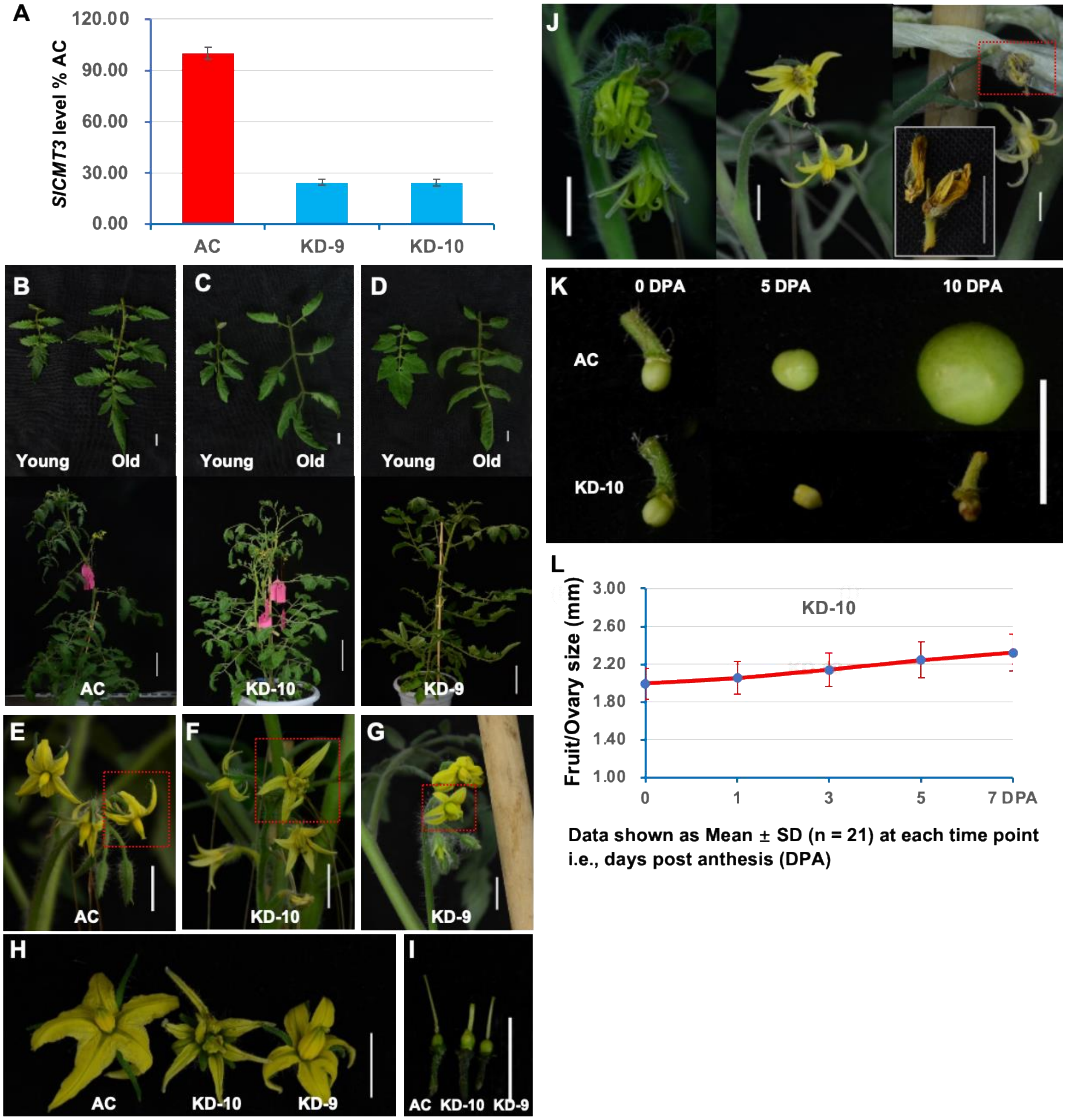

Fig. S2. Group-II *SlCMT3*-KD lines. (**A**) Reduction of *SlCMT3* expression in two independent Group-II *SlCMT3*-KD transgenic lines KD-9 and KD-10. RNAi-mediated suppression of endogenous *SlCMT3* expression was analysed by RT-qPCR. 18S rRNA was used as the internal control. When compared to the AC control, the average level of endogenous *SlCMT3* mRNA transcripts (mean ± SD, n = 4 i.e., four biological replicates/plants per line) was markedly reduced in each of 2 *SlCMT3*-KD transgenic lines KD-9 and KD-10 ($P \leq 0.05$; Tukey's test). (**B-D**) Vegetative growth and abnormal leaf architecture in Group-II *SlCMT3*-KD transgenic lines. One-

week young (Young) and one-month old (Old) compound leaves were photographed for AC (B) and Group-II lines KD-10 (C) and KD-9 (D). Six-week-old plants were photographed to show vegetative growth among tomato plants of the AC control line (B) and the two Group-II lines (C, D). Bar = 1 cm or 10 cm for leaves (Top) or entire plants (Bottom), respectively. (**E-I**) Defected flowers in Group-II *SlCMT3*-KD lines. Clusters of floral buds and flowers at different developmental stages in the control AC line (E) and 2 Group-II lines (F, G). Abnormal stamens/anther cones were observed in both KD lines (F-H). Fully opened flowers (boxed) are enlarged to show detailed floral structures (H). Within these anomalous anther cones, the style length was significantly shortened when compared to the AC control (I). (**J**) Irregular flowers in Group-II *SlCMT3*-KD line KD-10. Photographs were taken at 2 days post anthesis (- 2 DPA), 3 and 8 DPA, from left to right, respectively. An enlarged inlet image shows the wilted flower and stalled ovary (boxed) at 8 DPA. (**K**) Fruit set and expansion. Early ovary death is seen. Fruits or ovaries collected from the control AC line and *SlCMT3*-KD line KD-10 were photographed at 0, 5 and 10-DPA respectively. Bar = 1 cm in Panels E-K. (**L**) Stalled fruit/ovary expansion in *SlCMT3*-KD line KD-10.

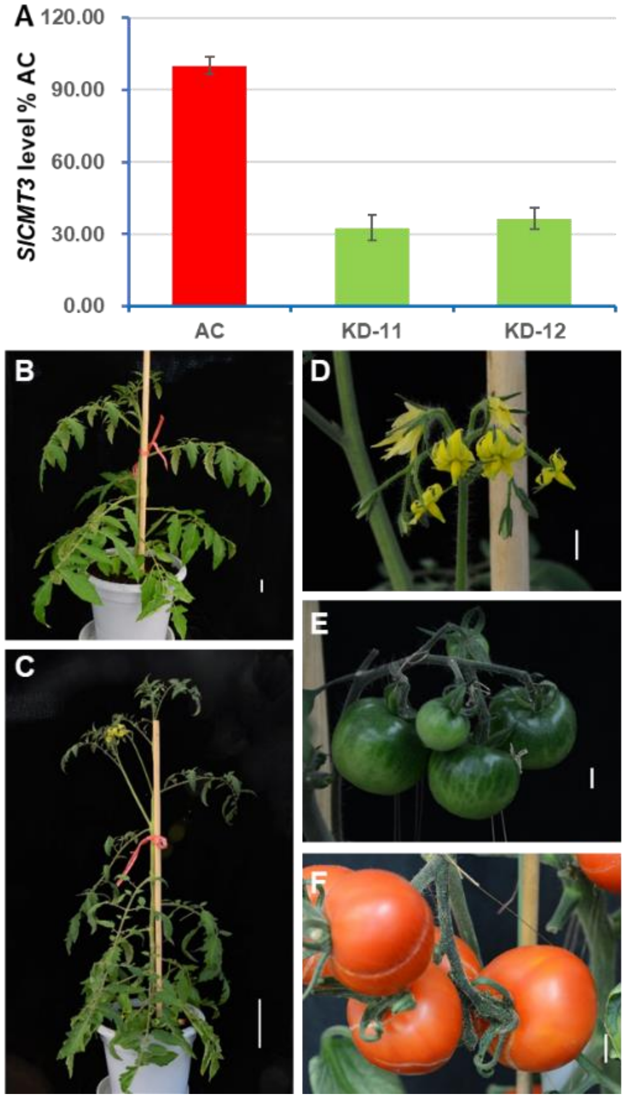

Fig. S3. *SlCMT3* RNAi has no effect on fruit development in Group-III *SlCMT3*-KD transgenic tomato lines. (**A**) Reduction of *SlCMT3* expression in two independent Group-III *SlCMT3*-KD transgenic lines. RNAi-mediated suppression of endogenous *SlCMT3* expression was analysed by RT-qPCR. 18S rRNA was used as the internal control. When compared to the AC control, the average level of endogenous *SlCMT3* mRNA transcripts (mean ± SD, n = 4, i.e., four biological replicates/plants per line) was markedly reduced in each of the two *SlCMT3*-KD transgenic lines KD-11 and KD-12 ($P \leq 0.05$; Tukey's test). (**B, C**) Normal vegetative growth. Three- (B) and six- (C) week old KD-11 plants. (**D**) Normal development of flowers in KD-11 plants. (**E, F**) Normal fruit development and ripening. Immature and mature green or red ripe fruits were photographed at 20-35 DPA (E) or 45-55 DPA (F) in KD-11 plants, respectively. Bar = 1 cm in (B) and (D-F), 10 cm in (C).

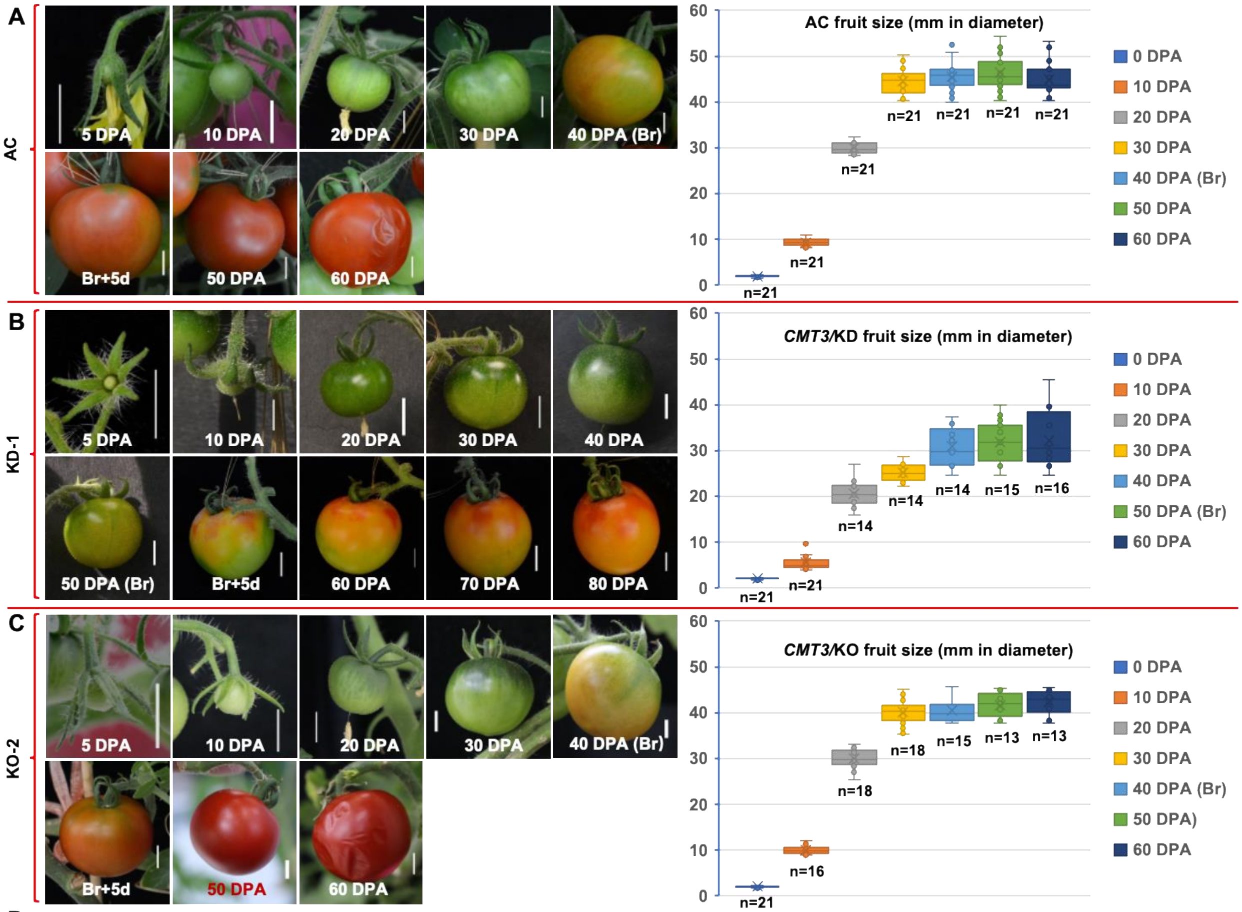

D Student's *t*-test for fruit size – p value

DPA	AC vs KD-1	AC vs KO-2	KD-1 vs KO-2
0	1.14E-01	7.65E-01	1.32E-01
10	8.61E-12	1.71E-02	2.28E-11
20	7.42E-15	9.58E-01	9.03E-12
30	3.92E-21	1.76E-05	5.36E-17
40	1.11E-13	4.79E-06	3.72E-08
50	1.69E-12	1.91E-04	1.23E-07
60	1.87E-09	2.13E-02	3.69E-06

Fig. S4. Impact of *SlCMT3* knockdown or knockout on tomato development and fruit ripening. (**A-C**) Fruit development and ripening. Fruits at different developmental and ripening stages were shown for the AC control line (A), *SlCMT3*-KD (KD-1; B) and *SlCMT3*-KO (KO-2; C) lines. DPA, days post anthesis; Br, breaker stage. Bar = 1 cm. Diameters of 13-21 fruits were measured at each DPA and data (mean ± SD (n = 13–21)) are shown at the left of each panel for AC, KD-1 and KO-2, respectively. (**D**) P-value. Student's ***t***-tests were performed on the AC control vs KD or KO line, and on KD vs KO.

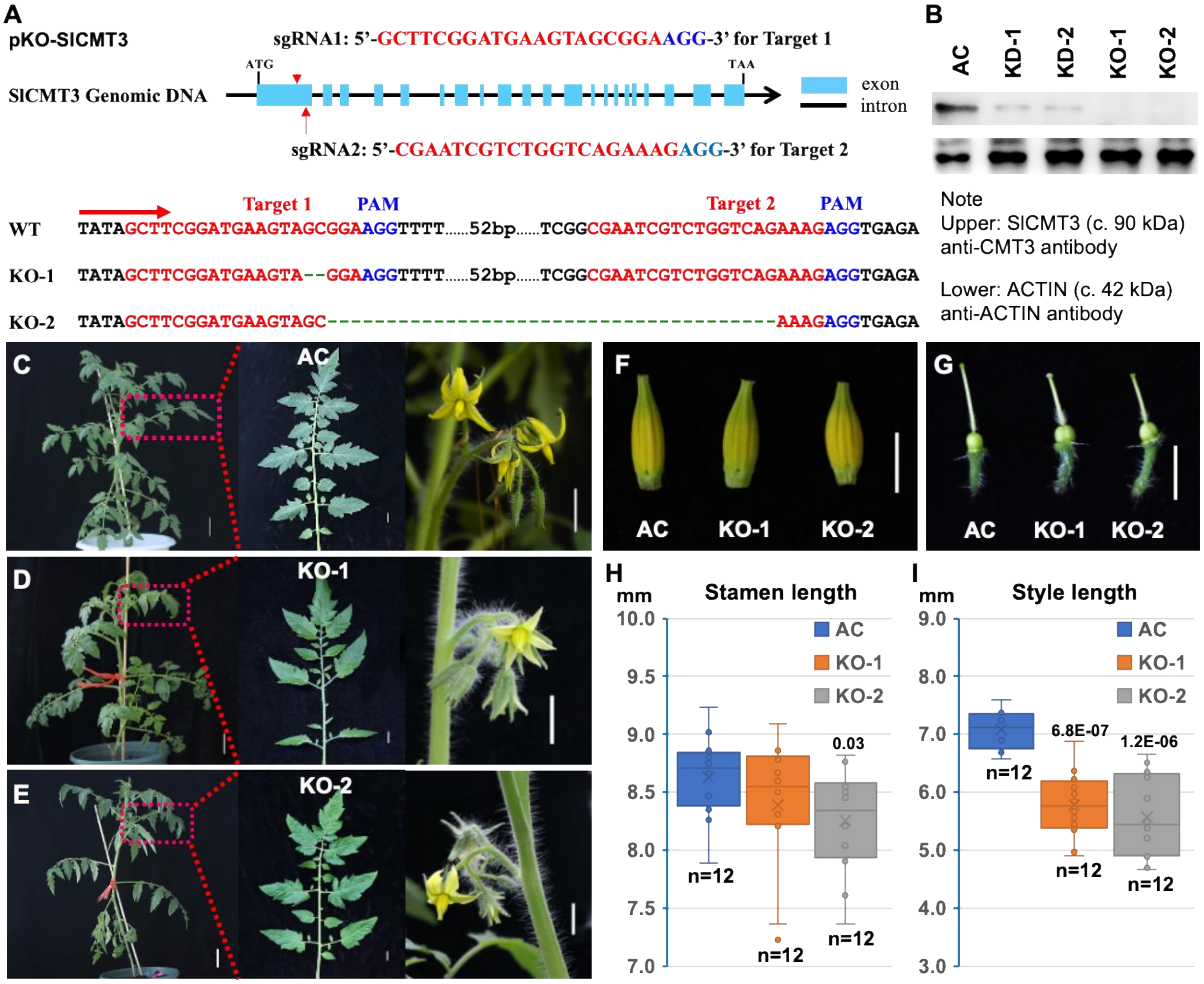

Fig. S5. CRISPR/Cas9-mediated *SlCMT3*-KO transgenic tomato lines. (**A**) Construction of the *SlCMT3*-knockout binary vector pKO-SlCMT3. Genomic organization of the *SlCMT3* gene includes the start and stop codon, exons (green box) and introns (solid black line segments) (Data set S1). The locations of sgRNA1 and sgRNA2 targets for CRISPR/Cas9-mediated gene editing are indicated (red arrow). Both sgRNA target sequences and related PAM sites are shown. Among nine *SlCMT3*-KO lines, two homozygous knockout lines KO-1 and KO-2 are shown here. KO-1 possesses a 2-bp deletion at the sgRNA1 target site, while KO-2 contains an 82-bp deletion from sgRNA1 to sgRNA2 target site. (**B**) Western blot detection of SlCMT3 protein. Total proteins extracted from anthers of AC, two *SlCMT3*-KO lines (KO-1 and KO-2) as well as two *SlCMT3*-KD lines (KD-1 and KD-2; fig. S1) were gel-separated and probed by anti-SlCMT3 antibody (upper panel), or anti-Actin antibody to show comparable loading of proteins (lower panel). Compared to the level of SlCMT3 protein in AC, no SlCMT3 protein was detectable in KO-1 and KO-2 although SlCMT3 was reduced in both KD-1 and KD-2. The molecular weights of SlCMT3 and Actin are

noted. (**C-G**) Impact of *SlCMT3* knockout on plant growth and leaf and flower development. In panels C-E, normal vegetative growth of 5-to-6 weeks old plants (left), fully developed normal (C) and abnormal (D, E) compound leaves (middle) and a cluster of flowers at different developmental stages (right) were shown for AC (C) and the two *SlCMT3*-KO lines KO-1 (D) and KO-2 (E). Similar morphology of stamen (anther cone, F) and style (G) among AC and 2 *SlCMT3*-KO lines KO-1 and KO-2. Bar = 5 cm (whole plant) or 1 cm (leaf and flower) in panels C-E, and 5 mm in panels F and G, respectively. (**H, I**) Effect of *SlCMT3*-KO on stamen (anther cone) and style length. Lengths of stamens (H) and styles (I) were measured on 12 fully opened flowers at 0-DPA collected from the AC control line and two *SlCMT3*-KO lines KO-1 and KO-2. Data are shown as mean ± SD (n = 12). Student's ***t***-test was performed on the AC control vs each individual KD line, and p-values are shown.

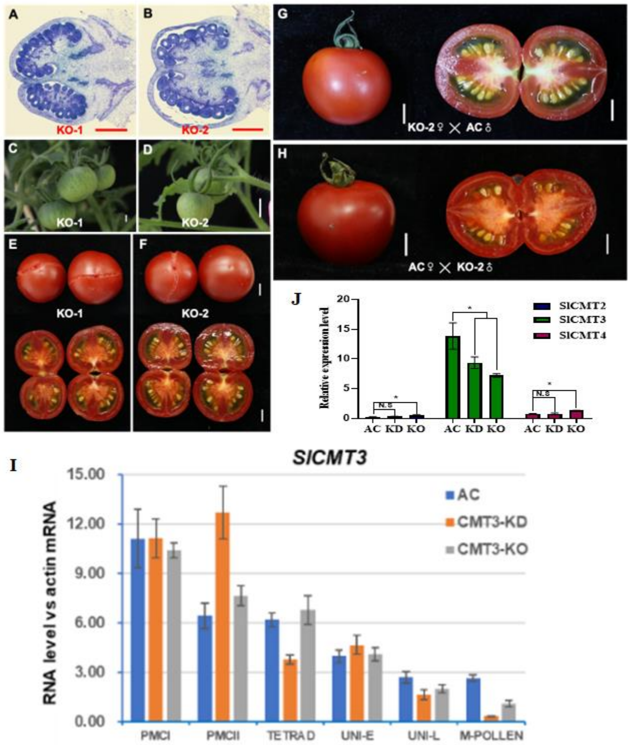

Fig. S6. Impact of *SlCMT3*-KO on fruit set, tomato development and ripening, and seed production. (**A, B**) Early seed formation. Normal fruit set and early seed formation are not affected in *SlCMT3*-KO lines KO-1 (A) and KO-2 (B). (**C-F**) Fruit development and ripening, and mature seed production. Intact or dissected tomato fruits from KO-1 (C, E) or KO-2 (D, F) were photographed at immature/mature green stage (10-25 DPA; C, D) or fully ripe fruits at 10 days after breaker (E, F). Seeds fully developed and matured in ripe fruits from both KO lines (lower panel in E, F). (**G, H**) Genetic crosses between AC and KO lines. Reciprocal crosses as indicated in G and H show that *SlCMT3*-KO does affect seed production. **I-J** The expression levels of three SlCMT genes in AC, KD and KO lines. Asterisk indicate statistical differences. N.S means No significance. Bar = 500 µm in A and B; 1 cm in C-H.

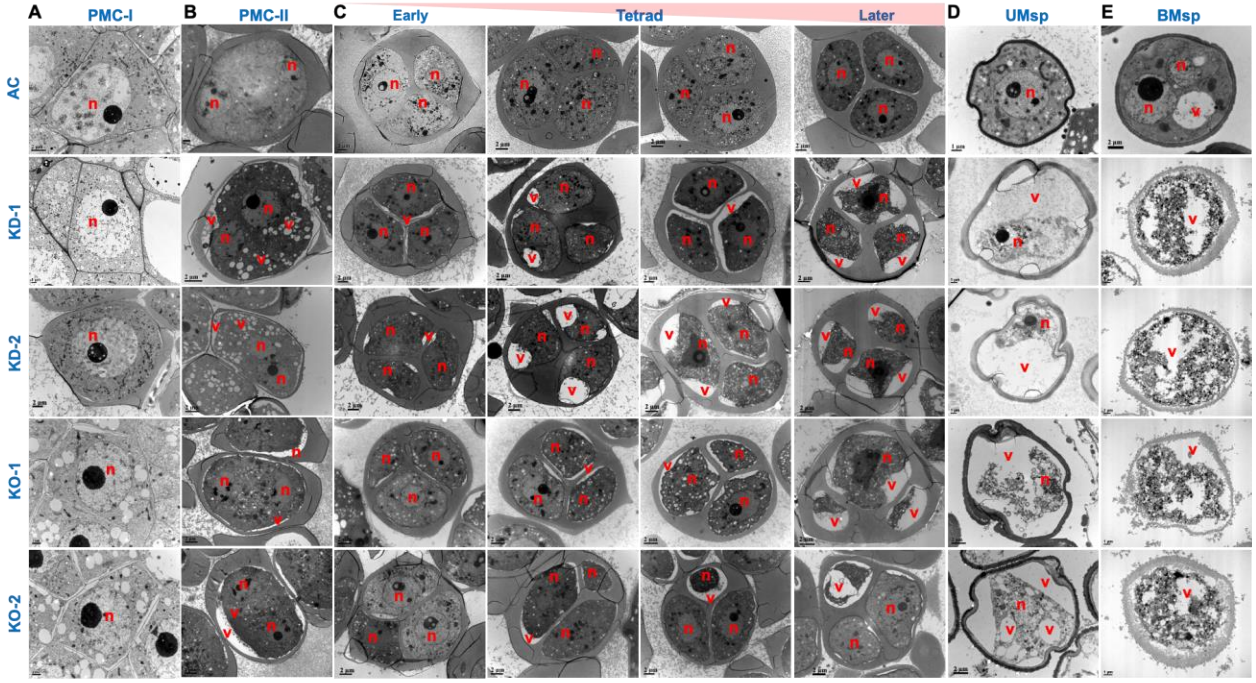

Fig. S7. Impact of *SlCMT3* on pollen development at ultrastructural level. (**A**) Pollen mother cell (PMC) at PMC stage I (PMC-I; prior to 1st meiosis). Large nuclei and concentrated cytoplasm are seen in AC; cytoplasm vacuolation can be seen in the two SlCMT3-KD lines KD-1 and KD-2, and also in the two SlCMT3-KO lines KO-1 and KO-2 KO. (**B**) PMC at PMC stage II (PMC-II; prior to 2nd meiosis). In AC, nucleus and cytoplasm divided into two. In KD and KO lines, uneven nuclear cytokinesis occurred in PMCs, and nucleus also divided. Cytoplasmic vacuolation started and numerous small vacuoles appeared in KD and KO lines, but less in AC. (**C**) Microspores at the tetrad stage (Tetrad, after 2nd meiosis). PMCs underwent two rounds of meiosis to produce four haploid microspores. In KD and KO, cytoplasm shrank, and cytoplasmic membrane and cell wall separated. Enlarged vacuoles were seen in KD and KO lines at the later Tetrad stage. (**D**) Microspore at the uninucleate microspore stage (UMsp). (**E**) Pollen cell at the late uninucleate/binucleate microspore stage (BMsp). Lower case letters n and v represent nucleus and vacuole. Bar = 2 μm.

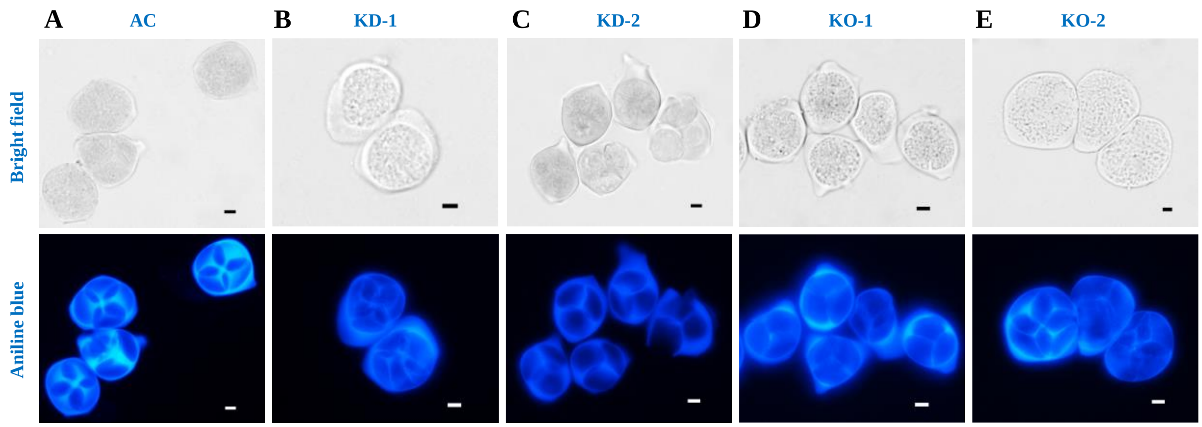

Fig.S8. Impact of *SlCMT3* on callose deposition during the tetrad stage. **(A-E)** Cytochemical staining for callose with aniline blue of tetrads from AC (A), two SlCMT3-KD lines KD-1 (B) and KD-2 (C), two SlCMT3-KO lines KO-1 (D) and KO-2 (E). In contrast to the normally deposited callose in the cell plate in AC, the aberrant decreased deposition of callose was observed at the periphery and intersporal walls in two KD and KO lines. Bar = 5 µm.

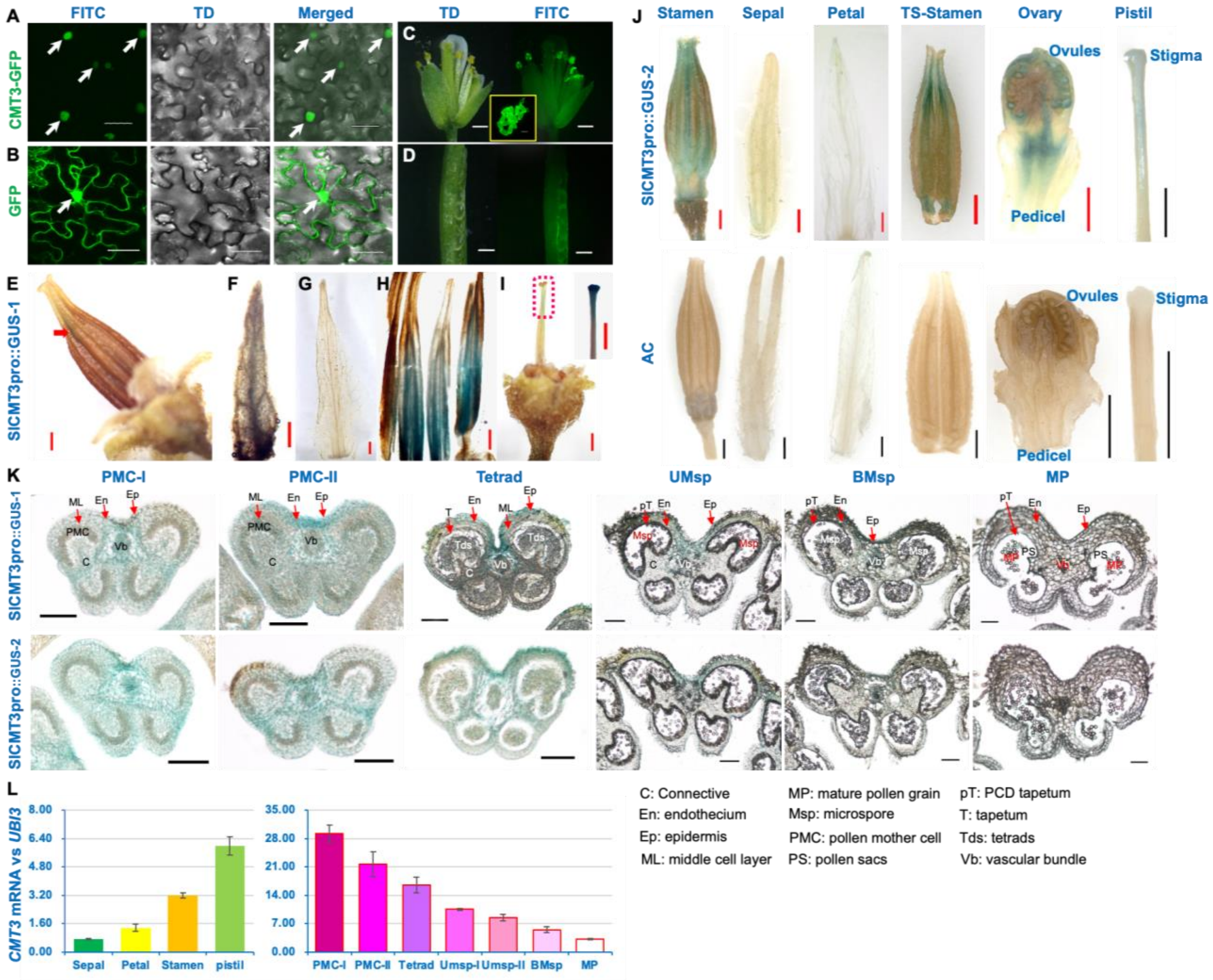

Fig. S9. Tissue-specific expression of nucleus-localized SlCMT3. (**A, B**) Nuclear localization of SlCMT3. Agroinfiltration assays shows that the SlCMT3-GFP fusion protein localises predominantly in nuclei (A). Free GFP is seen in cytoplasm, cell membrane and nuclei (B). Photographs were taken via FITC or TD channel. Merged images are also presented. Bar = 50 μm. (**C, D**) Tissue specific activity of the *SlCMT3* promoter in transgenic Arabidopsis Flowers (C) and seedpods (D) of homologous Arabidopsis *SlCMT3Pro:GFP* transgenic plants were photographed through TD bright field or FITC filter to show GFP green fluorescence as indicated. Strong fluorescent signals are predominantly present in stamens including mature pollen grains (inlet image; C), suggesting that the *SlCMT3* promoter activity is strong in these male tissues and cells. Bar = 500 μm in C and D, but 20 μm for the inlet image. (**E-J**) Activity of the *SlCMT3* gene promoter in different tomato tissues. Tomato floral tissues that were dissected from flowers of two *SlCMT3Pro:GUS* transgenic AC tomato lines as

indicated, GUS-stained and photographed through TD. Blue staining shows GUS expression under the control of the *SlCMT3* gene promoter. For SlCMT3Pro:GUS-1 (E-I) and SlCMT3Pro:GUS-2 (J), the intact stamen (anther cone, E), sepal (F), petal (G), longitudinal sections of stamen (LS stamen; H) and Pistil attached to ovary and an enlarged section (I) are shown. Blue staining in E is indicated by an arrow. Equivalent tissues collected from AC were used as GUS staining control as indicated. Bar = 1 mm. (**K**) Specific spatiotemporal *SlCMT3* promoter activity during pollen development. Anthers were collected from SlCMT3Pro:GUS-1 and SlCMT3Pro:GUS-2 lines at different developmental stages including PMC-I, PMC-II, Tetrad, uni- and bi-nucleate microspore (UMsp and BMsp), and mature pollen grain (MP), then GUS-stained, embedded, sectioned and photographed. Strong blue GUS staining was seen in various cell types at the PMC-I and PMC-II, but weakened at Tetrad > UMsp, and almost undetectable at BMsp and MP stages. Bar = 100 μm. (**L**) RT-qPCR assays - Spatiotemporal expression profiles of *SlCMT3* in floral organs and during pollen development. Left panel: *SlCMT3* expression pattern in the different organs of fully opened AC flowers as indicated. Right panel: *SlCMT3* expression pattern in stamens of AC floral buds at seven different stages corresponding to various pollen development as indicated. Stage I (PMC-I): floral bud < 2.5 mm (size in diameter); Stage II (PMC-II), floral bud ≅ 2.8-3.3 mm; Stage III (Tetrad), floral bud ≅ 3.5 - 4.5 mm; Stage IV (Umsp-I, early uninucleate microspore stage), floral bud ≅ 4.6 - 5.5 mm; Stage V (Umsp-II, late uninucleate microspore stage), floral bud ≅ 5.6 - 7.5 mm; Stage VI (Bmsp, binucleate microspore stage), floral bud ≅ 7.6 – 8.4 mm; Stage VII (MP, mature pollen stage), flower/bud ≥ 8.5 mm. *UBI3* was used as an internal control. Data are shown as mean ± SD (n = 3, i.e., three biological duplicates). One-way ANOVA was performed on each pair of adjacent samples, and all showed statistically significant differences ($P < 0.05$).

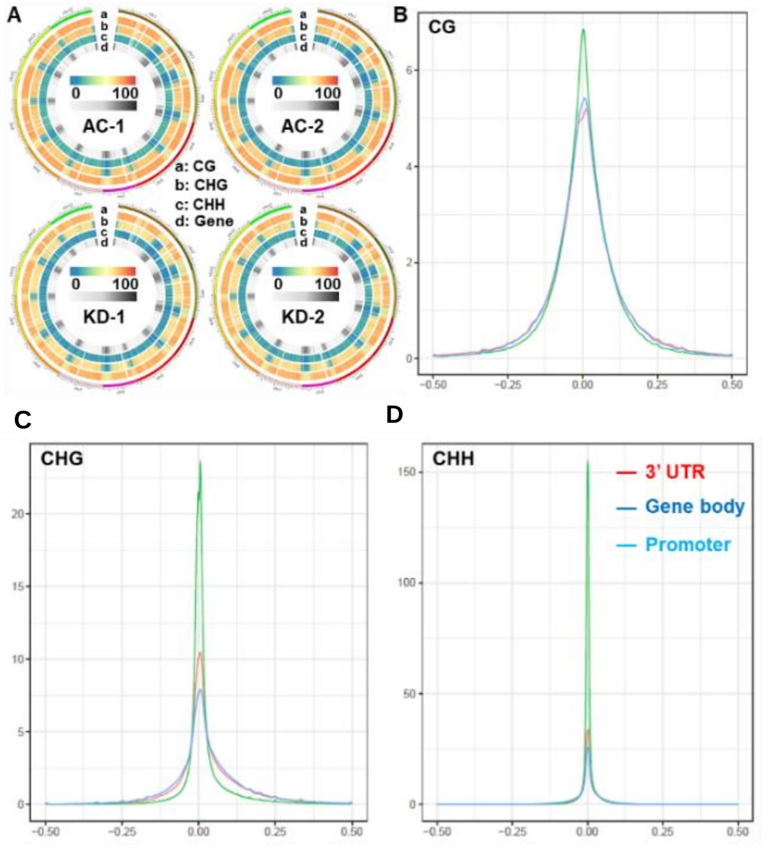

Fig. S10. Genome-wide DNA methylation. (**A**) Heatmap of expression profiles of DNA (de)methylation and maintenance genes in stamens. RNAseq analyses reveal that only *SlCMT3* and *SlMET1* were found to be up-regulated whilst *DML4* was down-regulated, others including *SlCMT2* were not affected in *SlCMT3*-KD lines KD-1 and KD-2 compared to AC controls AC-1 and AC-2. (**B-D**) Density plots of differential ^{m}CG (**B**), ^{m}CHG (**C**) and ^{m}CHH (**D**) between *SlCMT3*-KD lines and AC. Red, blue and green lines represent overall differential methylation levels at the 3'-UTR, gene body and promoter regions, respectively.

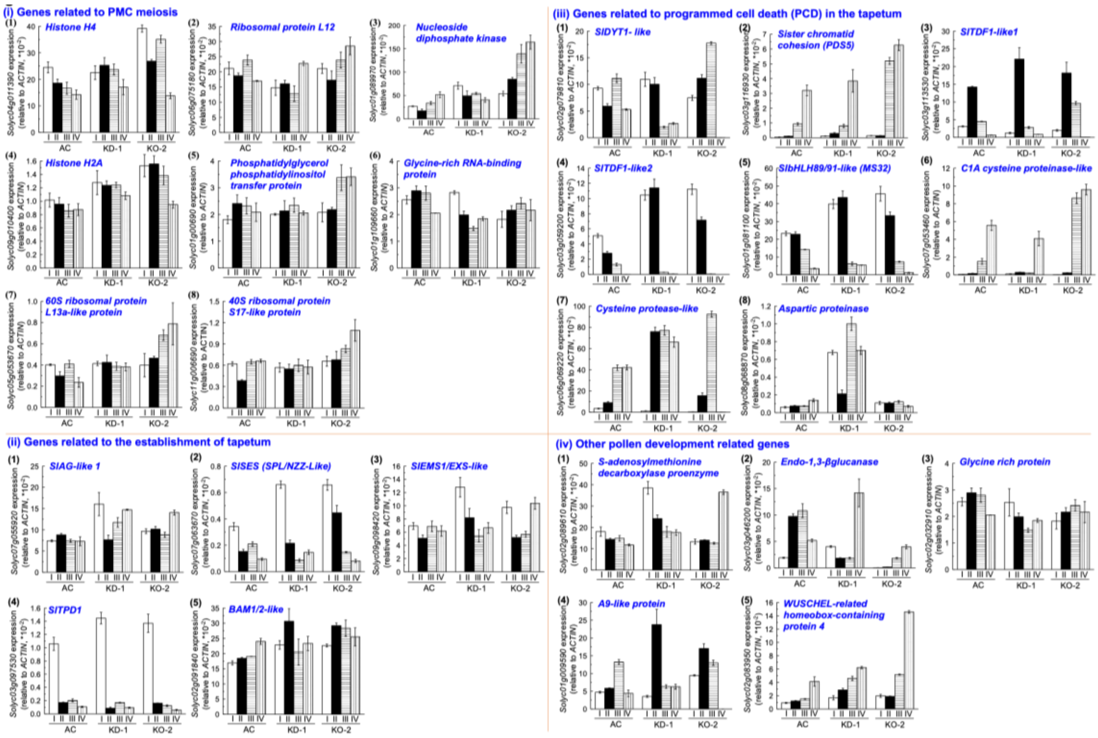

Fig. S11. Gene expression related pollen development in both KD and KO lines. (**i**) Genes related to PMC meiosis. (**ii**) Genes related to the establishment of tapetum. (**iii**) Genes related to tapetum programmed cell death (PCD). (**iv**) Other pollen development related genes. RT-qPCRs were performed on pollen mother cells or microspores at PMC-1 (I), PMC-II (II), Tetrad (III) and early uninucleate microspore (IV) stages. *ACTIN* was used as the internal control. Data are shown as mean ± SD (n = 3 i.e., three biological duplicates). One-way ANOVA was performed on AC vs SlCMT3-KD line KD-1 or on AC vs SlCMT3-KO line KO-2 at each corresponding pollen developmental stage.

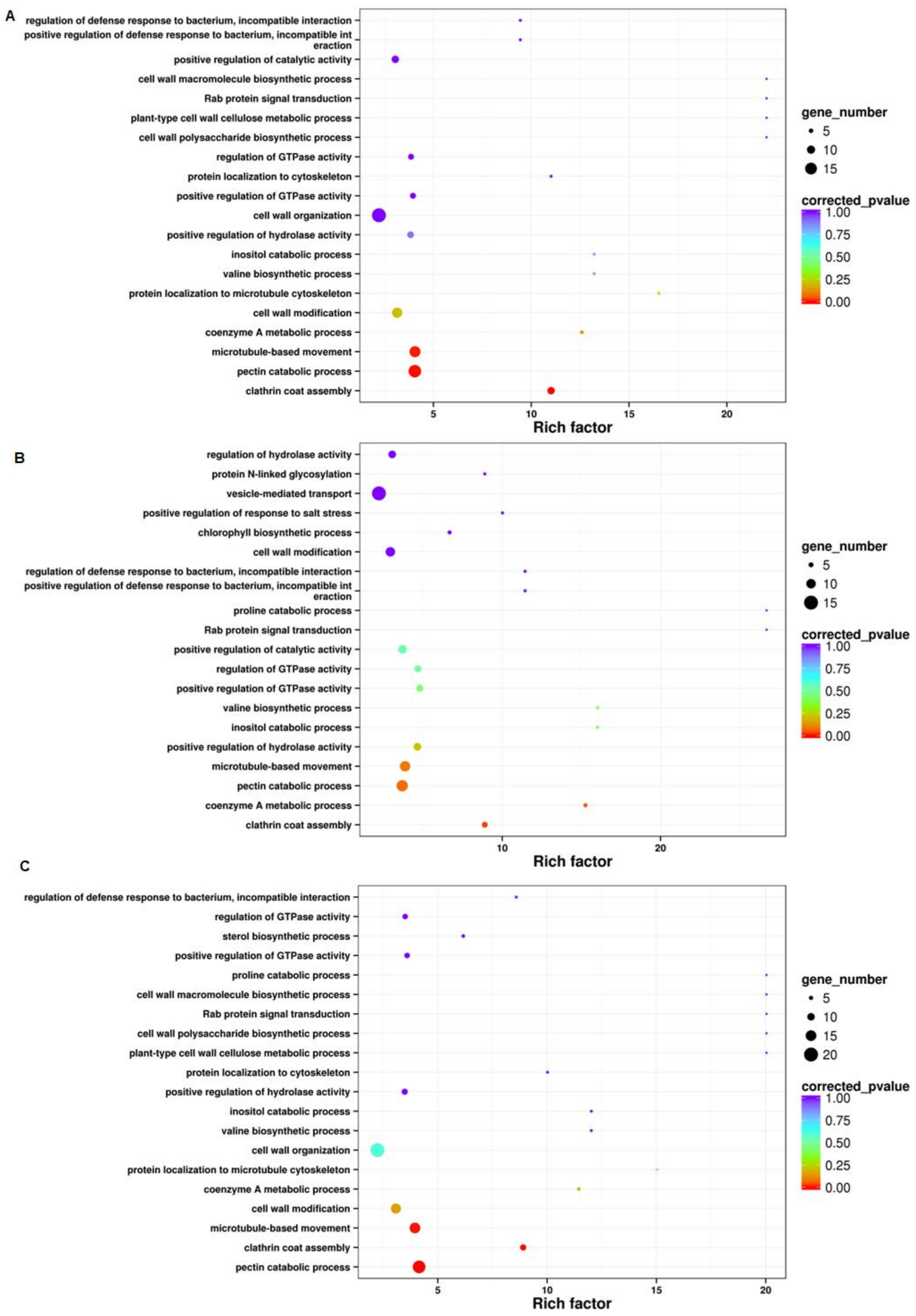

Fig. S12. GO enrichment analysis of the DEG^{DMR}s in CG, CHG and CHH context.
